# New Acridone- and (Thio)Xanthone-Derived 1,1-Donor–Acceptor-Substituted Alkenes: pH-Dependent Fluorescence and Unusual Photooxygenation Properties

**DOI:** 10.3390/molecules26113305

**Published:** 2021-05-31

**Authors:** Tim Lippold, Jörg M. Neudörfl, Axel Griesbeck

**Affiliations:** Department of Chemistry, Faculty of Natural Sciences and Mathematics, University of Cologne, Greinstr. 4-6, 50939 Köln-Cologne, Germany; lippoldt@smail.uni-koeln.de (T.L.); aco48@uni-koeln.de (J.M.N.)

**Keywords:** alkenes, acridones, singlet oxygen, fluorescence, Brønsted acid

## Abstract

A synthetic route to new heterocyclic 1,1-donor–acceptor-substituted alkenes starting with *N*-methyl-acridone, xanthone, and thioxanthone was investigated, leading to the acridone- and xanthone-derived products methyl 2-methoxy-2-(10-methylacridin-9 (10H)-ylidene)acetate (**7**) and methyl 2-methoxy-2-(9H-xanthen-9-ylidene)acetate (**10**) in low yields with the de-methoxylated product methyl 2-(10-methylacridin-9 (10H)-ylidene)acetate (**8**) and the reduced compound methyl 2-methoxy-2-(9H-xanthen-9-yl)acetate (**11**) as the major products from *N*-methyl acridone and xanthone. From thioxanthone, only the rearrangement and reduction products (**14**) and (**15**) resulted. The photophysical properties of compounds (**7**), (**8**), and (**10**) were investigated in the presence and absence of the Brønsted acid TFA by NMR, UV–VIS absorption, and fluorescence spectroscopy. Protonation of the acridone-derived alkenes (**7**) and (**8**) led to strong bathochromic and hyperchromic fluorescence shifts and a substantial increase in Stokes shift. The photooxygenation experiments with these substrates showed an unusual reactivity pattern in the singlet oxygen processes: whereas the electron-rich enolether (**7**) was chemically unreactive, (**8**) and (**10**) were oxidatively cleaved, presumably via intermediate 1,2-dioxetanes.

## 1. Introduction

**Scheme 1:**
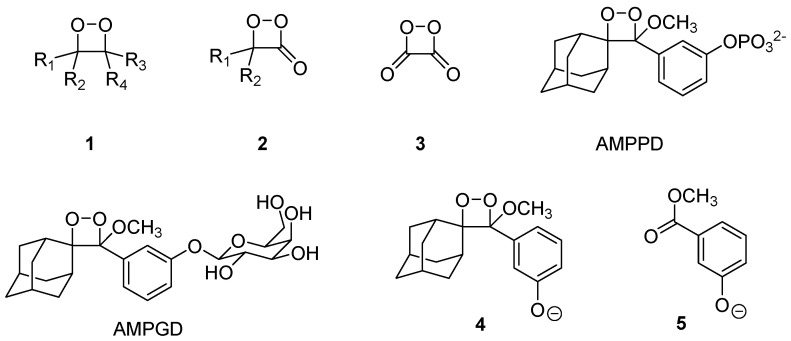
Structures of the three basic chemoluminescent peroxides: 1,2-dioxetanes (**1**), 1,2-dioxetanones (**2**), 1,2-dioxetanedione (**3**), and the CIEEL-active dioxetanes AMPPD and AMPGD and (**4**) with the fluorescent phenolate (**5**).

There are a very small number of unsaturated organic molecules that can be directly converted into four-membered cyclic peroxides by reaction with molecular singlet oxygen. These cyclic peroxides, such as 1,2-dioxetanes (**1**), 1,2-dioxetanones (**2**), and also 1,2-dioxetan-3,4-dione (**3**) (Scheme 1), represent the most potent compound class that can release bright and intensive light emission that appears in nature and in the laboratory as bio- and chemiluminescence (CL) [1,2]. The biological phenomena are known to mankind at least since three and a half millennia and can be traced back to ancient writings of Asian civilizations mentioning “fireflies” and “glow worms” for the first time. The Greek philosopher Aristotle mentioned the bioluminescence of fungi, and later on, Pliny the Elder even described bioluminescent clams, jelly, and lantern fishes [3,4]. In 1877, the Polish chemist *Radziszewski* realized the first artificial organic chemiluminescence, including a cyclic peroxide as the decisive metastable intermediate, from an alkaline solution of lophine that reacts with molecular oxygen [5]. Subsequently, more and more publications and discoveries were made, from the synthesis and characterization of lucigenine [6] and luminol [7,8] to peroxyoxalate chemiluminescence [9,10,11], which is also used in commercial glow sticks. Concerning the substituents, the stability and properties of 1,2-dioxetanes can vary greatly [12,13]. In order to populate the emitting state of the carbonyl product, formed in the thermal decomposition process, the cleavage of the O−O bond represents the first and rate-limiting step [14,15,16]. For the unsubstituted 1,2-dioxetane, experimental and theoretical investigations resulted in 23.0 and 23.5 kcal mol^−1^, respectively, for the activation energy required for the initial ring opening [17]. Besides this rather high energy barrier for the cleavage of the O−O bond, most uncatalyzed decompositions of 1,2-dioxetanes are proven to have a very poor emission efficiency due to the preferred formation of triplet excited carbonyl products [18,19]. 

Taking into account the effective spin–orbit coupling (SOC), according to *El-Sayed’s* rule [20,21], during “entropic trapping” [17], excited triplet states are more stable than excited singlet states, explaining the preferred formation of triplet excited products after the fragmentation process. With these major problems, it is not surprising that there is a manageable quantity of efficient CL systems and compounds such as luminol and lucigenine that are still used in numerous applications of present times as they represent the optimum of their compound classes. Small changes on efficient possible CL systems may lead to a major loss of light emission, which immensely increases the difficulty of the search for new and promising compounds. The first successful thermal preparation of a 1,2-dioxetane was reported by *Kopecky* and *Mumford*, who described the synthesis of trimethyl-1,2-dioxetane [22,23]. The [2 + 2] cycloaddition of electron-rich olefins with singlet oxygen, however, is the method of choice for the synthesis of 1,2-dioxetanes. In 1972, *Wieringa* et al. published the synthesis of bis-adamantylideneoxetane, the first dioxetane completely stable at room temperature, via a [2 + 2] cycloaddition with singlet oxygen and adamantylidene adamantine [24]. This scientific breakthrough enabled new possible syntheses and modifications of spiroadamantyl-substituted dioxetanes, which were stable enough to be used for analytical purposes, such as immunoassays and enzyme activity. *Schaap* and *Bronstein* developed 1,2-dioxetanes with adamantyl groups such as AMPPD and AMPGD (Scheme 1), which could be activated through the use of enzymes or chemically by releasing the protection group (phosphate, monomeric sugars, etc.) [25,26,27]. After the formation of the electronically excited species, either direct chemiluminescence or transfer of energy to a fluorophore that emits its characteristic fluorescence is observed. 

In contrast to the aforementioned thermal decomposition, a different mechanism for the spiroadamantane-1,2-dioxetanes is the chemically initiated electron exchange luminescence (CIEEL) [2,28,29]. Initiated through liberation of the phenolate by chemical or enzymatic agents, an excellent electron donor in the dioxetane (**4**) is generated. An intramolecular electron transfer from the phenolate into the O−O bond causes cleavage of this bond with the formation of a biradical anionic species. Finally, the simultaneous back electron transfer (BET) and C−C bond cleavage lead to the loss of adamantanone and the formation of the phenolate-substituted ester (**5**) in its excited singlet state [13,30,31,32]. The return to the singlet ground state is accompanied by emission of a photon (λ = 477 nm) [30]. The proposed CIEEL mechanism represents a recipe for effective chemiluminescence. The catalytic electron exchange weakens the O−O bond, thus decreasing the activation barrier and generating electronically excited singlet states for potential emission. However, the exact mechanism is still studied when concerning an intra- versus intermolecular BET [13,31]. Additionally, questioning the necessity of a full electron exchange, an alternative mechanism, the charge-transfer-induced luminescence (CTIL), has been proposed [33,34,35]. Besides this impressive and intensive research on chemi- and bioluminescence, there is still a demand for better CL systems for analytical and/or application purposes. We have recently published the first examples of donor–acceptor-substituted alkenes and their reactivity towards singlet oxygen [36]. The investigations and synthesis of new heterocyclic donor–acceptor-substituted alkenes as potential systems for chemoselective [2 + 2] cycloadditions with singlet oxygen are described herein and add a new approach to this complex topic.

## 2. Results

### 2.1. Synthesis of Donor–Acceptor Alkenes

**Scheme 2:**
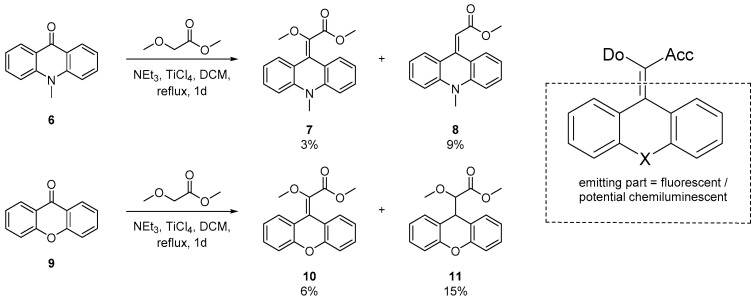
Synthesis of the donor–acceptor-alkenes (**7**) and (**10**) from coupling to acridone and xanthone with two unexpected side products, (**8**) and (**11**).

The 1,1-donor–acceptor-substituted alkenes were synthesized in one-pot procedures by applying a synthetic protocol by *Krick* et al. using a *Lehnert*-modified *Knoevenagel* condensation [37]. The olefination of 10-methylacridin-9(10H)-one (**6**) and xanthone (**9**) with methyl 2-methoxyacetate was successful using TiCl_4_-mediated α-methoxy methylacetate condensation with (**6**) and (**9**), respectively, albeit with low yields and with formation of by-products (**8**) and (**11**) (Scheme 2). Products (**7**) and (**10**) combine the two relevant features for potential chemiluminescent oxygenation processes: the electron-rich CC double bond that is reactive in singlet oxygen [2 + 2] cycloadditions and the oxidative cleavage products, acridone and xanthone, both highly fluorescent ketones. That this substitution pattern allows efficient dioxetane formation has already been shown for non-heterocyclic tetrasubstituted alkenes [36]. This synthetic approach delivers the desired target molecules, unfortunately in low yields, which is due, on the one hand, to moderate conversions (40%–50% in most cases) and, on the other hand, to the harsh reaction conditions that lead to two reductive pathways: reductive exchange of the 1-methoxy group by hydrogen leading to Michael ester product (**8**) and enone hydrogenation product (**11**) (and similar (**15**) from (**12**), vide infra).

**Scheme 3:**
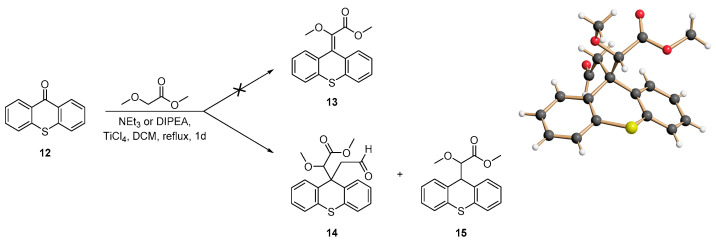
Conversion of the thioxanthone (**12**) into the double alkylation product (**14**) and the reduction product (**15**); structure of the aldehyde (**14**) in the crystal.

Applying the same synthesis protocol to thioxanthone (**12**) as starting material did not give the expected olefin (**13**) at all, but a mixture of the aldehyde (**14**) and the reduced derivative (**15**) (Scheme 3). The structurally unusual product (**14**) and the alkene (**15**) could also be analyzed by X-ray crystal structure determination (see SI). 

While the formation of (**15**) corresponds to an analogous reduction process as for (**11**), the formation and addition of the oxoethyl substituent of (**14**) seems cumbersome (but reproducible) and cannot be explained yet. In order to investigate whether a different amine base leads to different results, triethylamine (TEA) was exchanged by diisopropylethylamine (DIPEA). While the same products were obtained, their respective yields were higher, allowing a more efficient synthesis for these two compounds (**14**/**15**: 16% and 23% with TEA; 20% and 29% with DIPEA). In the case of the reaction with 10-methylacridin-9(10H)-one (**6**), the two alkenes (**7**) and (**8**) were obtained. 

In the case of the reaction with 10-methylacridin-9(10H)-one (**6**), the two olefins (**7**) and (**8**) were obtained. Here, a side reaction seems to occur, where the methoxy group of the α-carbon was reduced to a CH group, leading to the formation of the acridinylidene acetate. When comparing the results with those of *Krick* and *Lehnert*, who used rather strong α-acidic compounds, it appears that a reduction mechanism takes place in the reactions with weaker acids [37,38,39,40,41]. The deprotonation of methyl 2-methoxyacetate may also proceed slower and thus allows side reactions to occur. In all cases, the nitrogen base trimethylamine serves as the hydrogen donor in these reductive processes, which is also in agreement with the effect of base exchange, where better yields of these reduction products were obtained in the presence of DIPEA instead of TEA. 

### 2.2. Fluorescence and UV–VIS Absorption Properties

**Figure 1:**
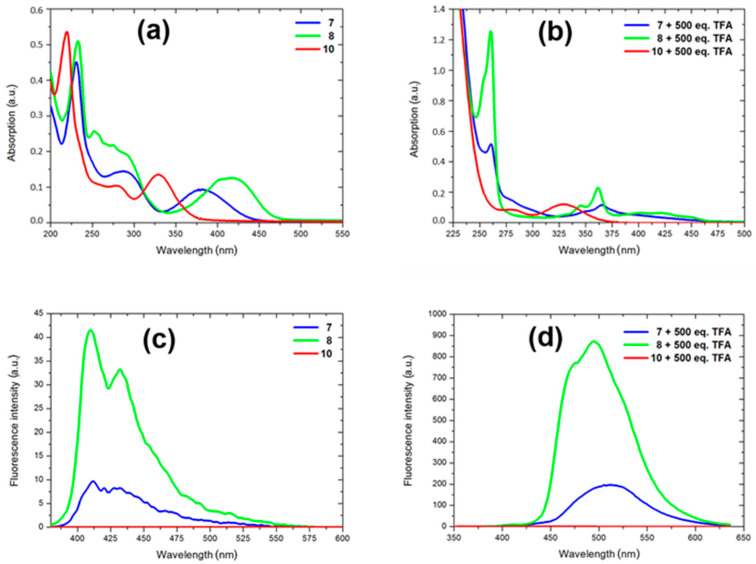
Absorption (**a**) and fluorescence (**c**) spectra of compounds (**7**) (blue), (**8**) (green), and (**10**) (red) in the absence of TFAin MeCN; absorption (**b**) and fluorescence (**d**) spectra after the addition of 500 eq. of TFA. Standardized compound concentration of 10^−5^ mol/L in MeCN.

The newly synthesized alkenes (**7**), (**8**), and (**10**) were characterized by fluorescence and UV–VIS spectroscopy (Figure 1 and Table 1, highlighted in yellow). Table 1 shows that these compounds are similar when comparing the absorption coefficients with system (**7**) having the lowest absorbance. While the xanthenylidene derivative (**10**) absorbs in the UV-A range with 329 nm, the absorption maxima of both acridinylidene derivatives (**7**) and (**8**) are shifted towards higher wavelengths of 378 and 417 nm, respectively. Of all three systems, only (**7**) and (**8**) show low fluorescence emission. The spectrum of (**8**) itself shows two distinguishable maxima at 410 and 432 nm. Even though the absorption maxima of the n → π* transitions of (**7**) and (**8**) can be seen at 378 and 417 nm, respectively, an excitation wavelength of 360 nm was chosen for the fluorescence spectra, which allowed the measurement of a spectrum where the complete curve progression was visible. 

Krick et al. performed TFA experiments with their acridinylidene systems and observed a proton-induced fluorescence [37]. Therefore, the heteroaromatic compounds (**7**), (**8**), and (**10**) were also measured in the presence 500 eq. of TFA to investigate whether a protonation occurs and how it influences the photophysical properties (Figure 1, Table 1). The absorption maxima of (**7**) and (**8**) were shifted towards shorter wavelengths, while the absorption spectrum of the xanthenylidene derivative (**10**) remained the same. Additional measurements in the presence of trifluoroacetic acid (TFA) were performed in order to gain information on the electronic structure of the conjugated π-system and to correlate fluorescence and absorption changes with protonation effects.

Every compound was measured with an identical concentration of c = 10^−5^ mol/L in acetonitrile to determine the absorption (λ_abs_) and emission (λ_em_) wavelength maxima, fluorescence quantum yields Φ_F_, and respective absorption coefficients ε^λ^. The fluorescence quantum yields Φ_F_ were determined comparatively using quinine hemisulfate in 0.1 M sulfuric acid as a reference substance [42].

**Figure 2:**
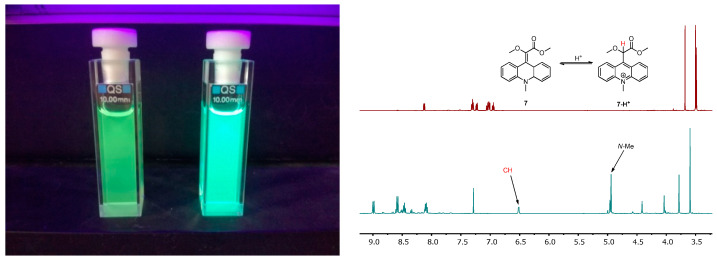
Left: solutions of acridinylidenes (**7**) and (**8**) (from left to right) in the presence of 500 eq. of TFA in MeCN. The samples were excited with an UV lamp (λ_em_ = 366 nm); right: acridinylidene (top) ^1^H-NMR of (**7**) without and (bottom) with 50 eq. of TFA. Two new significant signals at 4.92 (*N*-methyl group) and 6.50 ppm (strongly deshielded CH group) appear.

The addition of TFA shifts the absorption maxima for (**7**) and (**8**) to 366 and 362 nm, respectively, with additional blue-shifted maxima at 261 and 260 nm (Figure 1b). This suggests the possible formation of an acridinium cation, whose characteristic absorption maxima are at 260 nm and in the region of 300–500 nm [43,44]. Again, two maxima for the fluorescence, at 476 and 495 nm, can be seen in the curve progression of (**8**) (Figure 1d). While the emission wavelength values of (**8**) are in agreement with the reported emission maximum for N-methylacridinium (λ^em^_max_ = 490 nm) [45], compound (**7**) shows a red-shifted emission at 511 nm. Comparing the fluorescence intensities of the three substrates, compound (**10**) does not seem to react/interact with TFA so that under acidic conditions, the photophysical properties do not change. Acridinylidene systems (**7**) and especially (**8**) both show an increase in fluorescence characteristics after protonation with TFA (Figure 1d). The same observation was made when comparing the quantum yields. While the unprotonated substrates have weak or no fluorescence, a 30- to 40-fold increase in the quantum yields results from the addition of TFA (Table 1). For further investigation, 10 mg of each of acridinylidene derivatives (**7**) and (**8**) were dissolved in 1 mL CDCl_3_. Subsequently, 50 eq. of TFA was added to ensure a quantitative protonation, and 2D-NMR spectra were measured (^1^H-NMR: 500 MHz, ^13^C-NMR: 125 MHz) to determine the structure of the corresponding protonated species. Figure 2 shows the spectrum of compound (**7**) before and after the protonation. The two most significant changes are the new signals at 4.92 ppm with an integral of 3 protons (N-methyl group) and at 6.50 ppm with an integral of 1 proton (CH group of the geminal ether/ester moiety). Besides that, it can be seen that the proton signals in the aromatic region are shifted towards higher ppm values. 

Through the additional information of the 2D-NMR spectra, it could be proven that the protonation takes place at the carbon atom with the germinal ether/ester system and the acridinium cation (**7-H^+^**) is formed. This is in agreement with the results from a study by Krick et al. [18]. The NMR-measurements of (**8**) with 50 eq. TFA gave the same result (Figure 2). Comparing the ^1^H-NMR spectra before and after the protonation, a stronger deshielding effect in the aromatic region occurs. The spin systems consist of doublets and triplets, indicating that the aromatic protons became indistinguishable and formed a symmetric unit after the protonation. It is noteworthy that a CH_2_ fragment appears at 5.11 ppm and already hints that protonation occurred at the carbon atom of the olefin with the ester substituent. Again, with the investigation of all 2D-NMR spectra, it was proven that the acridinium cation (**8-H^+^**) was formed. Therefore, both systems show to be preferentially protonated at the α-carbon of the acceptor–donor moiety. The resulting formation of the acridinium cation increases the fluorescence intensity, especially in the case of compound (**8**). Comparing (**8-H^+^**) with the N-methyl acridinium cation of (**7-H^+^**), both values of the quantum yield and fluorescence intensity are remarkably higher.

### 2.3. Photooxygenation of the Geminal Acceptor–Donor Systems

The three alkenes (**7**), (**8**), and (**10**) were oxygenated with photosensitized singlet oxygen. Even though (**8**) does not represent an acceptor–donor system, it was also investigated to have a system comparable to (**7**). Every reaction was performed in an NMR tube with TPP as the photosensitizer (0.1 mg, c = 1.63 × 10^−4^ mol/L) in 1 mL of CDCl_3_. In general, 20 mg of the respective compounds were used. The reaction progress was monitored via ^1^H-NMR after constant time intervals.

**Figure 3:**
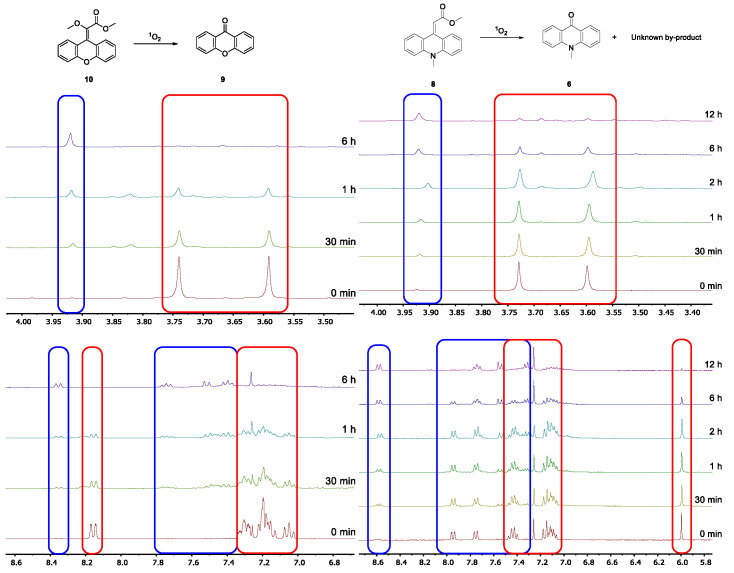
NMR time traces of photooxygenation experiment: xanthone-derived substrate (**10**) (**left**); acridone-derived substrate (**8**) (**right**). Blue boxes = increase of signal intensity; red boxes = decrease of signal intensity.

The photooxygenation of the xanthenylidene derivative (**10**) shows a decomposition reaction to the corresponding carbonyl compounds xanthone (7.39, 7.51, 7.74, and 8.35 ppm) and dimethyloxalate (3.92 ppm), suggesting a [2 + 2] cycloaddition with singlet oxygen (Figure 3). Comparing the integrals of dimethyloxalate with the signals of the methyl groups of the substrate (3.59 and 3.74 ppm) gives a distribution of 38% carbonyl products and 62% starting material after a reaction time of 1 h. The photooxygenation was performed for 6 h, but after 2.5–3 h, the decomposition was already quantitative.

The photooxygenation of other acridinylidene derivatives has already been proven to be a viable option for clearly visible chemiluminescence [46]. Investigation of the reactivity of the acridinylidene derivatives (**7**) and (**8**) towards singlet oxygen is therefore an interesting topic. While (**7**) possesses a geminal acceptor–donor structure, (**8**) only has an electron-withdrawing ester group that is expected to deactivate the carbon–carbon double bond in its reaction with singlet oxygen.

**Scheme 4:**
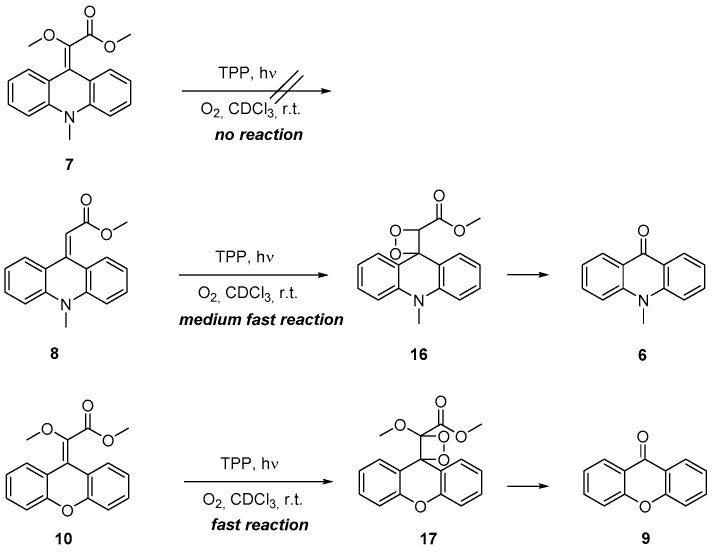
Photooxygenation of the acridone- and xanthone-derived alkenes (**7**), (**8**), and (**10**) and proposed 1,2-dioxetane intermediates (**16**) and (**17**).

Surprisingly, the methyl-protected acridinylidene derivative (**7**) did not react with singlet oxygen within the standard reaction time of 12 h (Scheme 4). The ^1^H-monitored photooxygenation of (**8**) is depicted in Figure 3, showing a quantitative decomposition to the corresponding carbonyl product N-methylacridone (**6**) with significant signals at 3.92, 7.31, 7.55, 7,74, and 8.58 ppm. The corresponding second cleavage product methyl 2-oxoacetate could not be detected in the NMR [47]. According to the literature, this compound easily polymerizes in solution [48].

Summarizing all photooxygenation experiments, three significant observations were made: (a) The 1,1-donor–acceptor-substituted alkene (**10**) behaves as expected for an electron-rich substrate without allylic hydrogens [36]. The formation of the carbonyl cleavage products indicates an initial [2 + 2] cycloaddition with ^1^O_2_ and subsequent cycloreversion of the dioxetane (**17**). (b) A similar reactivity was observed for the Michael substrate (**8**), which indicates that the lack of the electron-donating enolether group is compensated in part by the nitrogen-containing heterocycle that serves as an additional electron-donating group (as becomes also visible in the protonation experiments. (c) From observations (a) and (b), the conclusion is obvious that (**7**) must be the most reactive substrates because it combines enol ether and the tertiary amino group. This was not observed experimentally, quite the contrary. One explanation might be that conclusion (c) is nevertheless correct, but (**7**) serves as a strong physical singlet oxygen quencher as also known for other tertiary amines [49].

## 3. Materials and Methods

All reagents and solvents were purchased from commercial sources. The degree of purity of the compounds was at least 95%, and they were used without any further treatment. DCM was refluxed over calcium hydride under argon atmosphere and freshly distilled prior to use. ^1^H- and ^13^C-NMR spectra were recorded at 300 or 500 MHz and 75 or 125 MHz, respectively. The measurements were performed on a Bruker Avance III 500 MHz and on a Bruker Avance II 300 MHz (Bruker, Ettlingen, Germany). The chemical shifts *δ* are reported in ppm downfield of the internal standard of TMS [*δ* (^1^H-NMR) = 0.00 ppm, *δ* (^13^C-NMR) = 0.00 ppm]. CDCl_3_ [*δ* (^1^H-NMR) = 7.24 ppm, *δ* (^13^C-NMR) = 77.2 ppm] was used as the solvent. The coupling constant *J* is indicated in Hz. The fine structure is designated using the following abbreviations: s (singlet), d (doublet), t (triplet), q (quartet), quin (quintet), sxt (sextet), br (broad), and m (multiplet). Infrared spectra were measured with a Nicolet 380 FTIR (Thermo Fischer Scientific, Waltham, MA, USA). The wave numbers are categorized from 4000 to 800 cm^−1^. The signals are listed with the following abbreviations: w (weak), m (medium), s (strong), vs (very strong), and br (broad signal). The melting points of solid compounds were determined with a Melting Point apparatus B-545 (Büchi Labortechnik, Essen, Germany). Absorption spectra were measured with a UV–VIS spectrometer Type Lambda 35 (PerkinElmer, Waltham, MA, USA). Every sample was measured in quartz absorption cells with a diameter of 1 cm. Fluorescence spectra were measured with a luminescence spectrometer, LS-55B (PerkinElmer). Quartz fluorescence cells with a diameter of 1 cm were used for the measurements. High-resolution mass spectra were measured with a MAT 900 S and with an LTQ Orbitrap XL (Thermo Fischer Scientific, Waltham, MA, USA) via electrospray ionization (ESI). Crystal structure analyses were performed on a Nonius KappaCCD-Circle diffractometer. The structure was resolved using SHELXS-97 and SHELXL-97. Flash chromatography was performed on silica gel 60 Å, particle size 0.035–0.070 mm (Macherey-Nagel, Düren, Germany).

General procedure for olefination: In a double-heated three-necked flask with a reflux condenser and septum, TiCl_4_ (1.25 eq.) was slowly added under argon atmosphere to the ketone compound (1.00 eq.) dissolved in dry CH_2_Cl_2_ (15 mL per mmol ketone compound). The mixture was stirred for 15 min at room temperature. Methyl 2-methoxyacetate (2.00 eq.) and triethylamine or DIPEA (13.5 eq.) were added consecutively to the reaction mixture, which was subsequently heated under reflux for 24 h. An aqueous solution of HCl (1M) was added to the cooled-down reaction mixture until a clear solution emerged and extracted three times with CH_2_Cl_2_. The combined organic phases were washed with brine, dried over either MgSO_4_ or Na_2_SO_4_, and filtered, and the solvent was removed in vacuo. The residue was purified via column chromatography on SiO_2_.

General procedure for photooxygenation: In a NMR tube, the olefin (20 mg) was dissolved in CDCl_3_ (1 mL). An amount of 0.1 mg of TPP (c = 1.63 × 10^−4^ mol/L) was added, and the solution was saturated with oxygen over an inlet under light irradiation with two halogen lamps (150 W each) at 25 °C. The reaction progress was monitored via ^1^H-NMR after certain periods of time. After the reaction was completed, the product/products were not worked up or purified.

**Methyl 2-methoxy-2-(10-methylacridin-9(10H)-ylidene)acetate (7) and methyl 2-(10-methylacridin-9(10H)-ylidene)acetate (8)**

They were prepared according to the general procedure for olefinations, using 10-methylacridin-9(10H)-one (**6**) (1.00 g, 4.78 mmol, 1.00 eq.), methyl 2-methoxyacetate (0.95 mL, 9.56 mmol, 2.00 eq.), triethylamine (8.90 mL, 64.5 mmol, 13.5 eq.), TiCl_4_ (0.65 mL, 5.98 mmol, 1.25 eq.) in DCM (60 mL). The crude products were purified by flash column chromatography with *n*-hexane/EtOAc (18:1). Products (**7**) and (**8**) were both obtained as a green viscous oil with a yield of 0.04 g (0.14 mmol, 3%) and 0.12 g (0.43 mmol, 9%) respectively.

Analytical data for **7**: R_f_ = 0.36 (5:1, *n*-Hex/EtOAc). ^1^H-NMR (500 MHz, CDCl_3_, 298 K): δ [ppm] = 3.49 (s, 3H), 3.50 (s, 3H), 3.68 (s, 3H), 6.95 (td, *J* = 7.4 and 1.0 Hz, 1H), 7.01 (m, 1H), 7.03 (m, 1H), 7.05 (m, 1H), 7.23 (dd, *J* = 7.8 and 1.5 Hz, 1H), 7.30 (m, 2H), 8.13 (dd, *J* = 7.9 and 1.6 Hz, 1H). ^13^C-NMR (125 MHz, CDCl_3_, 298 K): δ [ppm] = 33.6 (CH_3_), 51.5 (CH_3_), 57.7 (CH_3_), 112.0 (CH_arom_), 112.3 (CH_arom_), 120.3 (CH_arom_), 120.4 (CH_arom_), 121.2 (C_q_), 121.4 (C_q_), 123.0 (C_q_), 127.4 (CH_arom_), 128.3 (CH_arom_), 128.6 (CH_arom_), 128.7 (CH_arom_), 139.1 (C_q_), 141.7 (C_q_), 142.1 (C_q_), 166.4 (C_q_). IR (FTIR): ῦ [cm^−1^] = 2943 (w), 2833 (w), 2361 (w), 1712 (s), 1592 (m), 1496 (w), 1463 (vs), 1431 (w), 1351 (m), 1333 (w), 1257 (vs), 1217 (m), 1190 (w), 1168 (w), 1152 (w), 1132 (w), 1115 (m), 1063 (w), 1051 (w), 1024 (w), 940 (w), 962 (w), 911 (w), 888 (w), 858 (w), 806 (w). HRMS (ESI): calcd. [M + H]^+^: 296.12811, found: 296.12873; calcd. [M + Na]^+^: 318.11006, found: 318.11049.

Analytical data for **8**: R_f_ = 0.27 (5:1, *n*-Hex/EtOAc). ^1^H-NMR (500 MHz, CDCl_3_, 298 K): δ [ppm] = 3.59 (s, 3H), 3.73 (s, 3H), 6.00 (s, 1H), 7.08 (m, 1H), 7.10 (m, 1H), 7.13 (m, 1H), 7.16 (m, 1H), 7.42 (m, 1H), 7.44 (m, 1H), 7.75 (dd, *J* = 7.9 and 1.5 Hz, 1H), 7.95 (dd, *J* = 7.9 and 1.5 Hz). ^13^C-NMR (125 MHz, CDCl_3_, 298 K): δ [ppm] = 34.0 (CH_3_), 51.1 (CH_3_), 108.6 (CH), 112.9 (CH_arom_), 113.1 (CH_arom_), 119.5 (CH_arom_), 119.9 (C_arom_), 121.4 (C_arom_), 124.0 (C_q_), 124.2 (CH_arom_), 129.8 (CH_arom_), 130.3 (CH_arom_), 130.6 (CH_arom_), 140.1 (C_q_), 141.4 (C_q_), 145.3 (C_q_), 167.6 (C_q_). IR (FTIR): ῦ [cm^−1^] = 2946 (w), 1703 (m), 1591 (s), 1487 (w), 1462 (vs), 1431 (w), 1356 (w), 1301 (w), 1269 (w), 1156 (vs), 1130 (vs), 1060 (w), 1049 (w), 971 (w), 938 (w), 888 (w), 856 (w). HRMS (ESI): calcd. [M + H]^+^: 266.11756, found: 266.11771; calcd. [M + Na]^+^: 282.11247, found: 282.11268.

**Methyl 2-methoxy-2-(9H-xanthen-9-ylidene)acetate (10) and methyl 2-methoxy-2-(9H-xanthen-9-yl)acetate (11)**

They were prepared according to the general procedure for olefinations, using xanthone (**9**) (0.94 g, 4.80 mmol, 1.00 eq.), methyl 2-methoxyacetate (1.00 mL, 9.61 mmol, 2.00 eq.), triethylamine (9.00 mL, 64.8 mmol, 13.5 eq.), TiCl_4_ (0.66 mL, 6.00 mmol, 1.25 eq.) in DCM (60 mL). The crude products were purified by flash column chromatography with *c*-hexane/EtOAc (20:1→10:1). Both separated products were then freed of remaining xanthone via Kugelrohr distillation (165 °C, 5.7 mbar). Product (**10**) was obtained as a yellow solid and (**11**) as a yellow viscous oil with yields of 0.08 g (0.28 mmol, 6%) and 0.20 g (0.70 mmol, 15%), respectively.

Analytical data for **10**: R_f_ = 0.18 (20:1, *c*-Hex/EtOAc). ^1^H-NMR (500 MHz, CDCl_3_, 298 K): δ [ppm] = 3.59 (s, 3H), 3.74 (s, 3H), 7.05 (ddd, *J* = 7.9, 7.2, and 1.3 Hz, 1H), 7.15 (m, 1H), 7.19 (m, 2H), 7.21 (m, 1H), 7.30 (m, 2H), 8.15 (dd, *J* = 8.0 and 1.6 Hz, 1H). ^13^C-NMR (125 MHz, CDCl_3_, 298 K): δ [ppm] = 51.9 (CH_3_), 57.6 (CH_3_), 116.2 (CH_arom_), 116.5 (CH_arom_), 117.1 (C_q_), 121.0 (C_q_), 121.4 (C_q_), 122.8 (CH_arom_), 123.1 (CH_arom_), 126.7 (CH_arom_), 128.7 (CH_arom_), 128.9 (CH_arom_), 129.1 (CH_arom_), 140.9 (C_q_), 152.3 (C_q_), 152.6 (C_q_), 165.9 (C_q_). IR (FTIR): ῦ [cm^−1^] = 2942 (w), 1716 (vs), 1656 (w), 1597 (w), 1581 (w), 1476 (m), 1450 (vs), 1337 (m), 1252 (vs), 1226 (vs), 1208 (s), 1161 (m), 1128 (s), 1109 (s), 1101 (s), 1043 (m), 1022 (s), 962 (w), 940 (w), 914 (w), 898 (w), 881 (w), 864 (w), 816 (w). M.p.: 72–75 °C. 

HRMS (ESI): calcd. [M + H]^+^: 283.09648, found: 283.09679; calcd. [M + Na]^+^: 305.07843, found: 305.07868.

Analytical data for **11**: R_f_ = 0.07 (20:1, *c*-Hex/EtOAc). ^1^H-NMR (500 MHz, CDCl_3_, 298 K): δ [ppm] = 3.21 (s, 3H), 3.60 (s, 3H), 3.83 (d, *J* = 5.6 Hz, 1H), 4.40 (d, *J* = 5.6 Hz, 1H), 7.07 (tt, *J* = 7.5 and 1.7 Hz, 2H), 7.11 (d, *J* = 7.9 Hz, 1H), 7.12 (d, *J* = 7.9 Hz, 1H), 7.22 (dd, *J* = 7.6 and 1.4 Hz, 1H), 7.24 (m, 1H), 7.26 (m, 1H), 7.28 (m, 1H). ^13^C-NMR (125 MHz, CDCl_3_, 298 K): δ [ppm] = 43.1 (CH), 51.8 (CH_3_), 59.0 (CH_3_), 86.5 (CH), 116.4 (CH_arom_), 116.5 (CH_arom_), 120.8 (C_q_), 121.0 (C_q_), 123.0 (CH_arom_), 123.2 (CH_arom_), 128.4 (CH_arom_), 128.5 (CH_arom_), 128.8 (CH_arom_), 129.4 (CH_arom_), 152.8 (C_q_), 153.0 (C_q_), 171.2 (C_q_). IR (FTIR): ῦ [cm^−1^] = 2949 (w), 2831 (w), 1748 (m), 1651 (w), 1601 (w), 1577 (w), 1458 (s), 1355 (w), 1301 (w), 1254 (vs), 1201 (m), 1154 (w), 1118 (s), 1027 (w), 1003 (w), 963 (w), 942 (w), 927 (w), 893 (w), 861 (w), 830 (w). HRMS (ESI): calcd. [M + Na]^+^: 307.09408, found: 307.09427.

**Methyl 2-methoxy-2-(9-(2-oxoethyl)-9H-thioxanthen-9-yl)acetate (14) and methyl 2-methoxy-2-(9H-thioxanthen-9-yl)acetate (15)**

They were prepared according to the general procedure for olefinations, using thioxanthone (**12**) (1.00 g, 4.71 mmol, 1.00 eq.), methyl 2-methoxyacetate (0.93 mL, 9.42 mmol, 2.00 eq.), DIPEA (10.8 mL, 63.6 mmol, 13.5 eq.), TiCl_4_ (0.65 mL, 5.89 mmol, 1.25 eq.) in DCM (60 mL). The crude products were purified by flash column chromatography with *n*-hexane/EtOAc (4:1). Product (**14**) was obtained as a colorless solid with a camphor-like smell and (**15**) as an orange solid with yields of 0.32 g (0.93 mmol, 20%) and 0.41 g (0.43 mmol, 29%), respectively.

Analytical data for **14**: R_f_ = 0.23 (4:1, *n*-Hex/EtOAc). ^1^H-NMR (500 MHz, CDCl_3_, 298 K): δ [ppm] = 2.99 (s, 3H), 3.27 (s, 3H), 3.71 (dd, *J* = 19.7 and 2.3 Hz, 1H), 3.97 (dd, *J* = 19.7 and 1.9 Hz), 4.48 (s, 1H), 7.19 (m, 1H), 7.22 (m, 1H), 7.26 (m, 1H), 7.29 (m, 1H), 7.30 (m, 1H), 7.41 (m, 1H), 7.48 (m, 2H), 9.61 (t, *J* = 2.0 Hz, 1H). ^13^C-NMR (125 MHz, CDCl_3_, 298 K): δ [ppm] = 40.5 (CH_2_), 48.9 (C_q_), 51.6 (CH_3_), 58.9 (CH_3_), 78.5 (CH), 126.5 (CH_arom_), 126.6 (CH_arom_), 127.1 (CH_arom_), 127.2 (CH_arom_), 127.3 (CH_arom_), 127.4 (CH_arom_), 127.7 (CH_arom_), 128.9 (CH_arom_), 131.9 (C_q_), 133.1 (C_q_), 133.3 (C_q_), 134.4 (C_q_), 170.6 (C_q_), 202.1 (C_aldehyde_). IR (FTIR): ῦ [cm^−1^] = 3583 (w), 3564 (w), 3445 (w), 2924 (w), 2852 (w), 2360 (w), 2343 (w), 1738 (vs), 1717 (vs), 1464 (m), 1445 (w), 1425 (w), 1362 (w), 1311 (w), 1301 (w), 1272 (w), 1219 (m), 1197 (s), 1176 (w), 1113 (s), 1064 (w), 1051 (w), 1012 (w), 956 (m), 945 (w), 913 (w), 811 (w). M.p.: 131.4 °C. HRMS (ESI): calcd. [M + H]^+^: 343.09985, found: 343.09992; calcd. [M + Na]^+^: 365.08180, found: 365.08170.

Analytical data for **15**: R_f_ = 0.36 (4:1, *n*-Hex/EtOAc). ^1^H-NMR (500 MHz, CDCl_3_, 298 K): δ [ppm] = 3.10 (s, 3H), 3.42 (s, 3H), 4.13 (d, *J* = 9.7 Hz, 1H), 4.36 (d, *J* = 9.6 Hz, 1H), 7.17 (m, 1H), 7.19 (m, 1H), 7.21 (m, 1H), 7.23 (m, 1H), 7.25 (m, 1H), 7.40 (m, 1H), 7.42 (m, 2H). ^13^C-NMR (125 MHz, CDCl_3_, 298 K): δ [ppm] = 51.6 (CH_3_), 52.6 (CH), 58.6 (CH_3_), 79.0 (CH), 126.3 (CH_arom_), 126.4 (CH_arom_), 126.6 (CH_arom_), 126.9 (CH_arom_), 127.2 (CH_arom_), 127.4 (CH_arom_), 129.4 (CH_arom_), 131.3 (CH_arom_), 132.2 (C_q_), 132.8 (C_q_), 133.4 (C_q_), 134.1 (C_q_), 171.8 (C_q_). IR (FTIR): ῦ [cm^−1^] = 3059 (w), 2996 (w), 2934 (w), 2832 (w), 2359 (w), 2343 (w), 1744 (vs), 1585 (w), 1465 (m), 1445 (m), 1430 (w), 1365 (w), 1313 (w), 1295 (w), 1277 (w), 1243 (w), 1227 (w), 1203 (s), 1176 (m), 1151 (w), 1113 (s), 1078 (w), 1065 (w), 1041 (w), 1030 (w), 1007 (m), 982 (w), 961 (w), 947 (w), 928 (w), 887 (w), 873 (w), 860 (w), 830 (w). HRMS (ESI): calcd. [M + Na]^+^: 323.07123, found: 323.07155.

Photooxygenation of methyl 2-(10-methylacridin-9(10H)-ylidene)acetate (**8**).

The reaction was performed according to the general procedure for photooxygenation using (**8**) (20 mg, 0.07 mmol, 1.00 eq.) and TPP (1.0 mg, c = 1.63 × 10^−4^ mol/L) dissolved in CDCl_3_ (1 mL). After 12 h, the reaction was completed, and 10-methoxyacridin-9(10H)-one (**19**) and an unidentifiable compound were obtained.

Photooxygenation of methyl 2-methoxy-2-(9H-xanthen-9-ylidene)acetate (**10**)

The reaction was performed according to the general procedure for photooxygenation using (**10**) (20 mg, 0.07 mmol, 1.00 eq.) and TPP (1.0 mg, c = 1.63 × 10^−4^ mol/L) dissolved in CDCl_3_ (1 mL). After 6 h, the reaction was completed, and xanthone (**9**) and dimethyloxalate were obtained.

## 4. Conclusions

Two new heterocyclic donor–acceptor-substituted alkenes (**7**) and (**10**) were synthesized via *Knoevenagel* condensation from 10-methylacridin-9(10H)-one (**6**) and xanthone (**9**) with titanium (IV) chloride. An additional acceptor-substituted compound (**8**) that does not represent an acceptor–donor system was also included in the photooxygenation study. The results of the singlet oxygen reactions showed that (**8**) and (**10**) are versatile substrates for singlet oxygen [2 + 2] cycloaddition reactions, whereas (**7**) surprisingly does not show chemical quenching abilities, possibly an effect of a strong physical quenching of singlet oxygen by the tertiary amine (**7**). Concerning their photophysics, the heteroaromatic compounds (**7**) and (**8**) were only slightly fluorescent and (**10**) showed no fluorescence at all. After the addition of 500 eq. of TFA, however, a strong increase in fluorescence intensity was detected for (**7**) and (**8**), while the xanthenylidene derivative (**10**) did not change in fluorescence. NMR analyses revealed the formation of the corresponding acridinium cation structures (**7**H^+^) and (**8**H^+^).

## Figures and Tables

**Table 1:** Low-energy (red-shifted) absorption wavelengths (λ^abs^_max_), corresponding absorption coefficients (ε), excitation wavelengths (λ^ex^), emission maxima (λ^em^_max_), and fluorescence quantum yields Φ_F_ of (**7**), (**8**), and (**10**) without (−) and with (+) 500 eq. of TFA. The data highlighted in yellow are from the experiments without acid added.

Compound	TFA	λ^abs^_max_ (nm)	Absorption Coefficient ε^λ^ (L/mol·cm)	λ^ex^ (nm)	λ^em^_max_ (nm)	Quantum Yield Φ_F_
7	−	378	9370	360	398	0.008
	+	366	11,580	366	511	0.310
8	−	417	12,540	360	410/432	0.035
	+	362	22,890	362	476/495	0.886
10	−	329	13,520	329	−	−
	+	329	12,040	329	−	−

## Data Availability

Data are contained within the article or Supplementary Material.

## Supplementary Materials

The following are available online: NMR and IR spectra of compounds (**7**), (**8**), (**10**), (**11**), (**14**), and (**15**); X-ray data of compounds (**14**) and (**15**) [50].
