# Supplementary material for: New Acridone- and (Thio)Xanthone-Derived 1,1-Donor–Acceptor-Substituted Alkenes: pH-Dependent Fluorescence and Unusual Photooxygenation Properties
Source: Molecules. 2021 May 31;26(11):3305. doi: 10.3390/molecules26113305 (PMC8198218; doi:10.3390/molecules26113305)
Supplement: Supplementary file 1 [file molecules-26-03305-s001.zip › molecules-1219467-supplementary.pdf]

Supporting Information

New acridone and (thio)xanthone-derived 1,1-donor-acceptor-substituted alkenes: pH-dependent fluorescence and unusual photooxygenation properties

Tim Lippold, Jörg M. Neudörfl,
Axel G. Griesbeck
Department of Chemistry,
University of Cologne

Author Contributions

A.G.G. Conceptualization: Lead; Funding acquisition: Lead
T.L. Investigation: Lead
J.M.N. Methodology: Lead

Supporting Information

Table of contents

1. NMR and IR spectra 1

1.1 NMR and IR spectra of compound 7 1

1.2 NMR and IR spectra of compound 8 4

1.3 NMR and IR spectra of compound 10 7

1.4 NMR and IR spectra of compound 11 10

1.5 NMR and IR spectra of compound 14 13

1.6 NMR and IR spectra of compound 15 16

2. X-ray data 19

2.1 Data of X-ray crystal measurement of 14 19

2.2 Data of X-ray crystal measurement of 15 20

1. NMR and IR spectra

1.1 NMR and IR spectra of compound 7

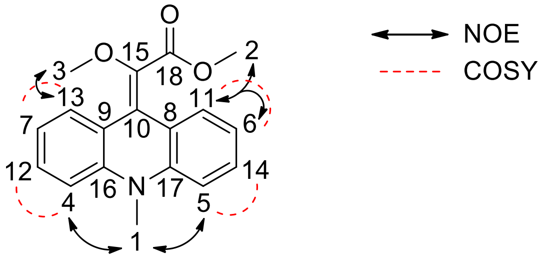

Table S1: 1D and 2D-NMR data of methyl-2-methoxy-2-(10-methylacridin-9(10H)-ylidene)acetate (7) in $CDCl_3$, at 298 K and 500 MHz for ^1H and 125 MHz for ^{13}C.

No.	δ_H (*J* in Hz)	δ_C, mult.	HMBC (^xJ)
1	3.49 (3H, s)	33.6, CH_3	-
2	3.68 (3H, s)	51.5, CH_3	-
3	3.50 (3H, s)	57.7, CH_3	-
4	7.01 (1H, m)	112.0, CH_{arom}	C4→H1(^4J), H7(^3J), H12(^2J), H13(^4J)
5	7.03 (1H, m)	112.3, CH_{arom}	C5→H1(^4J), H6(^3J), H11(^4J), H14(^2J)
6	6.95 (1H, td, 7.4/1.0 Hz)	120.3, CH_{arom}	C6→H5(^3J)
7	7.05 (1H, m)	120.4, CH_{arom}	C7→H4(^3J)
8		121.2, C_q	C8→H5(^3J), H6(^3J)
9		121.4, C_q	C9→H4(^3J), H7(^3J)
10		123.0, C_q	C10→H11(^3J), H13(^3J)
11	7.23 (1H, dd, 7.8/1.5 Hz)	127.4, CH_{arom}	C11→H14(^3J)
12	7.30 (1H, m)	128.3, CH_{arom}	C12→H13(^3J)
13	8.13 (1H, dd, 7.9/1.6 Hz)	128.6, CH_{arom}	C13→H12(^3J)
14	7.30 (1H, m)	128.7, CH_{arom}	C14→H11(^3J)
15		139.1, C_q	C15→H2(^3J), H3(^4J)
16		141.7, C_q	C16→H1(^3J), H7(^4J), H12(^3J), H13(^3J)
17		142.1, C_q	C17→H1(^3J), H6(^4J), H11(^3J), H14(^3J)
18		166.4, C_q	C18→H2(^3J)

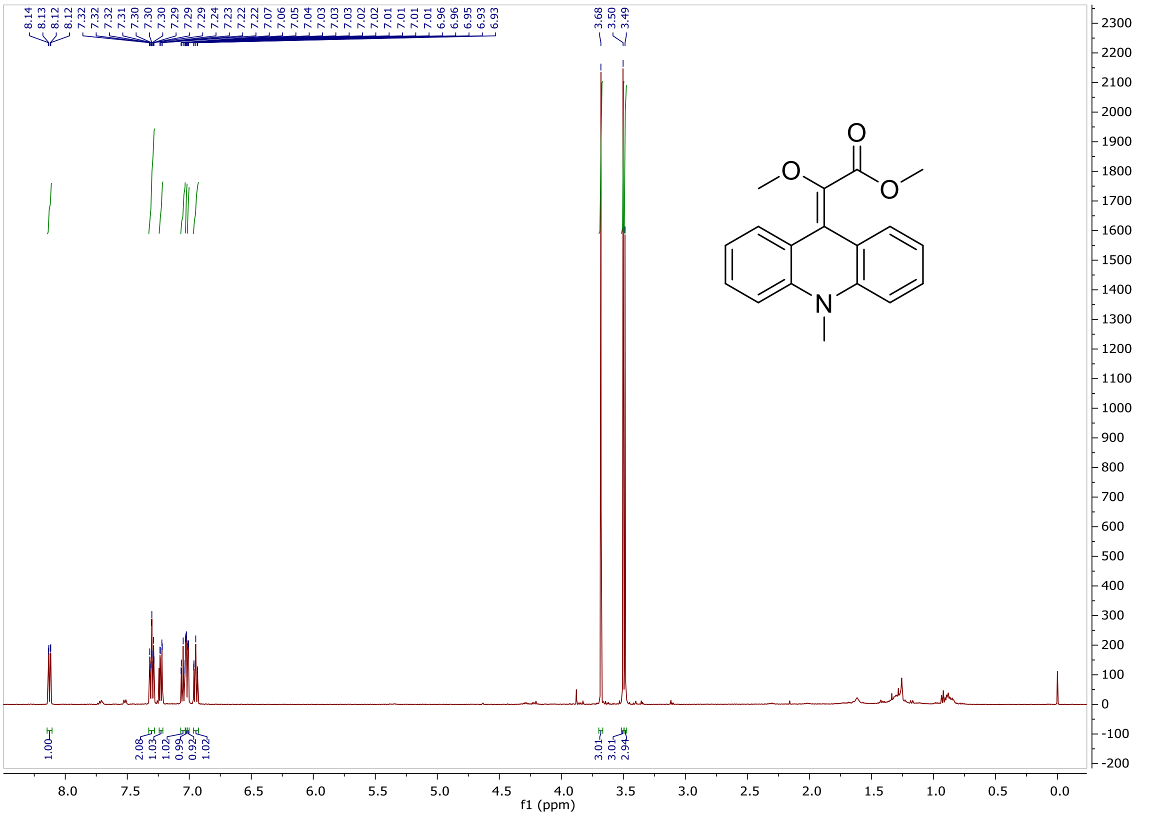
8.14
8.13
8.12
8.12
7.32
7.32
7.32
7.31
7.30
7.30
7.30
7.29
7.29
7.29
7.24
7.23
7.22
7.22
7.07
7.06
7.05
7.04
7.03
7.03
7.03
7.02
7.02
7.01
7.01
7.01
7.01
6.96
6.96
6.95
6.93
6.93
3.68
3.50
3.49
1.00
2.08
1.03
1.02
0.99
0.92
1.02
3.01
3.01
2.94
O
O
O
N
f1 (ppm)

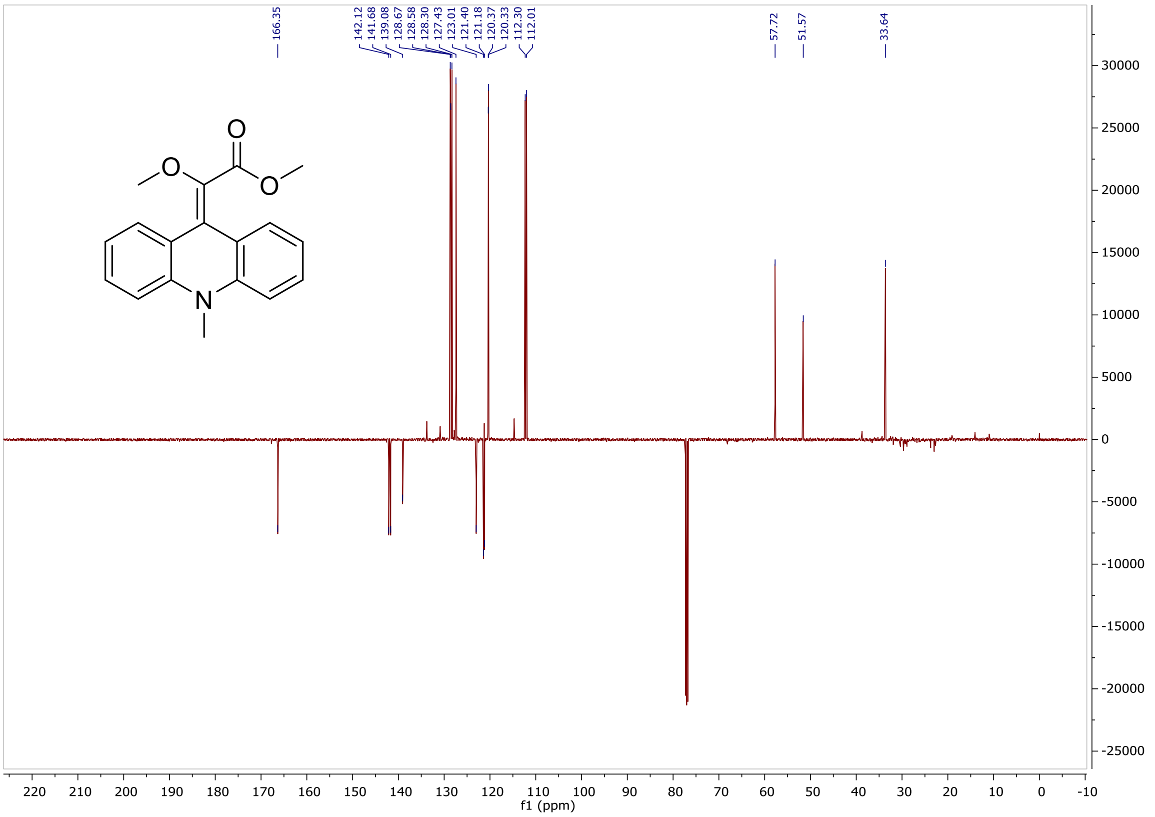
166.35
142.12
141.68
139.08
128.67
128.58
128.30
127.43
123.01
121.40
121.18
120.37
120.33
112.30
112.01
57.72
51.57
33.64
O
O
O
N
f1 (ppm)

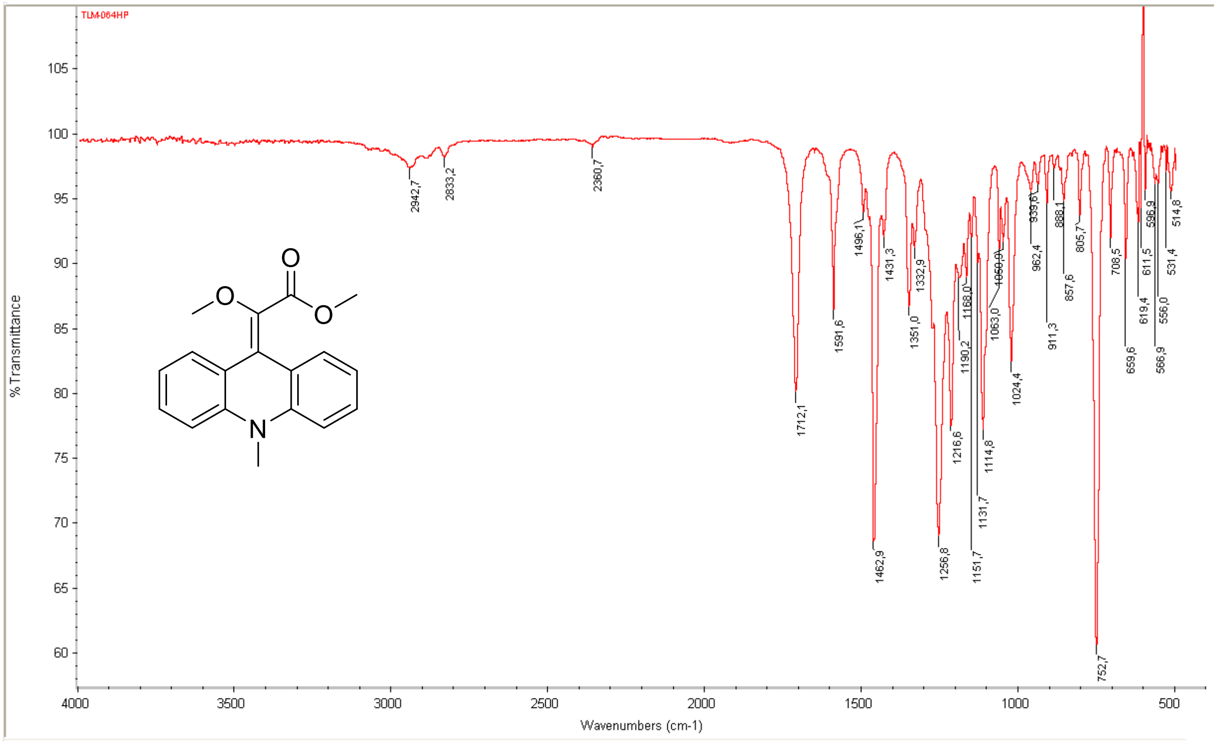

Figure S1: NMR- and IR-spectra of **7**.

1.2 NMR and IR spectra of compound 8

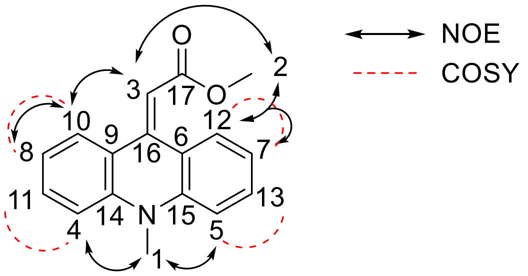

<u>Table S2:</u> 1D and 2D-NMR data of Methyl 2-(acridin-9(10H)-ylidene)acetate (8) in $CDCl_3$, at 298 K and 500 MHz for ^{1}H and 125 MHz for ^{13}C.

No.	δ_H (*J* in Hz)	δ_C, mult.	HMBC (^{x}J)
1	3.59 (3H, s)	34.0, CH_3	-
2	3.73 (3H, s)	51.1, CH_3	-
3	6.00 (1H, s)	108.6, CH	C3→H2(^{4}J)
4	7.13 (1H, m)	112.9, CH_{arom}	C4→H1(^{5}J), H8(^{3}J), H10(^{4}J), H11(^{2}J)
5	7.16 (1H, m)	113.1, CH_{arom}	C5→H1(^{5}J), H7(^{3}J), H12(^{4}J), H13(^{2}J)
6		119.5, C_q	C6→H3(^{3}J), H7(^{3}J)
7	7.08 (1H, m)	119.9, CH_{arom}	C7→H5(^{3}J)
8	7.10 (1H, m)	121.4, CH_{arom}	C8→H4(^{3}J)
9		124.0, C_q	C9→H3(^{3}J), H8(^{3}J)
10	7.75 (1H, dd, 7.9/1.5 Hz)	124.2, CH_{arom}	C10→H11(^{3}J)
11	7.42 (1H, m)	129.8, CH_{arom}	C11→H10(^{3}J)
12	7.95 (1H, dd, 7.9/1.5 Hz)	130.3, CH_{arom}	C12→H13(^{3}J)
13	7.44 (1H, m)	130.6, CH_{arom}	C13→H12(^{3}J)
14		140.1, C_q	C14→H1(^{4}J), H8(^{4}J), H10(^{3}J), H11(^{3}J)
15		141.4, C_q	C15→H1(^{4}J), H7(^{3}J), H12(^{3}J), H13(^{3}J)
16		145.3, C_q	C16→H3(^{2}J), H4(^{4}J), H5(^{4}J), H10(^{3}J), H12(^{3}J)
17		167.6, C_q	C17→H2(^{3}J)

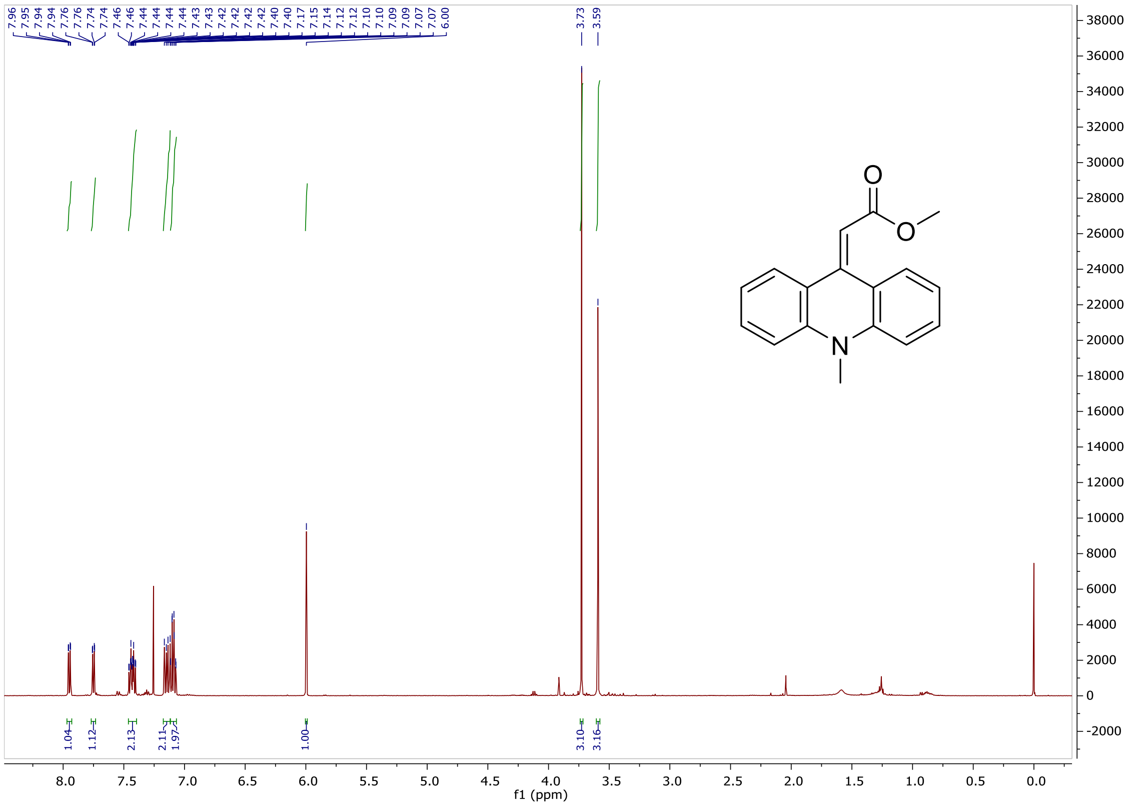
7.96 7.95 7.94 7.94 7.76 7.76 7.74 7.74 7.46 7.46 7.44 7.44 7.44 7.44 7.43 7.43 7.42 7.42 7.42 7.42 7.40 7.40 7.40 7.17 7.15 7.14 7.12 7.12 7.10 7.10 7.09 7.09 7.07 7.07 6.00
3.73
3.59
1.04 1.12 2.13 2.11 1.97 1.00 3.10 3.16
O
O
N
f1 (ppm)

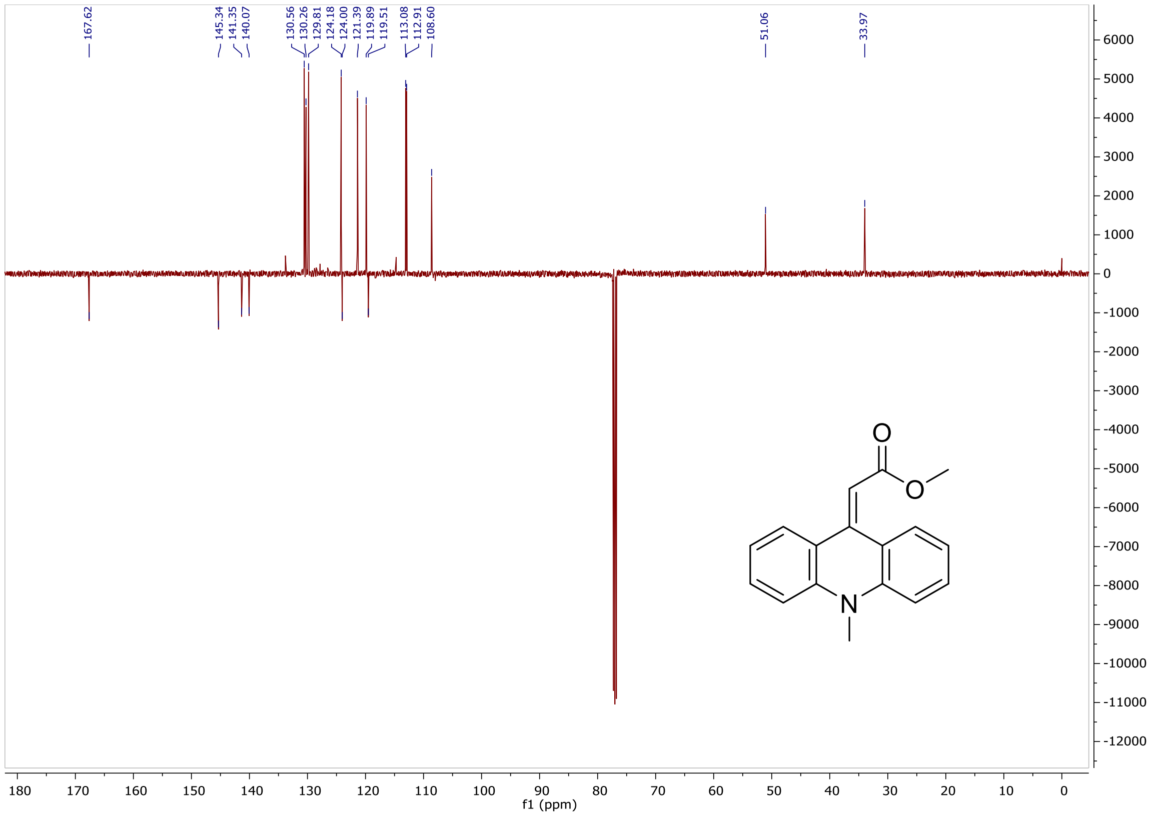
167.62
145.34 141.35 140.07
130.56 130.26 129.81 124.18 124.00 121.39 119.89 119.51 113.08 112.91 108.60
51.06
33.97
O
O
N
f1 (ppm)

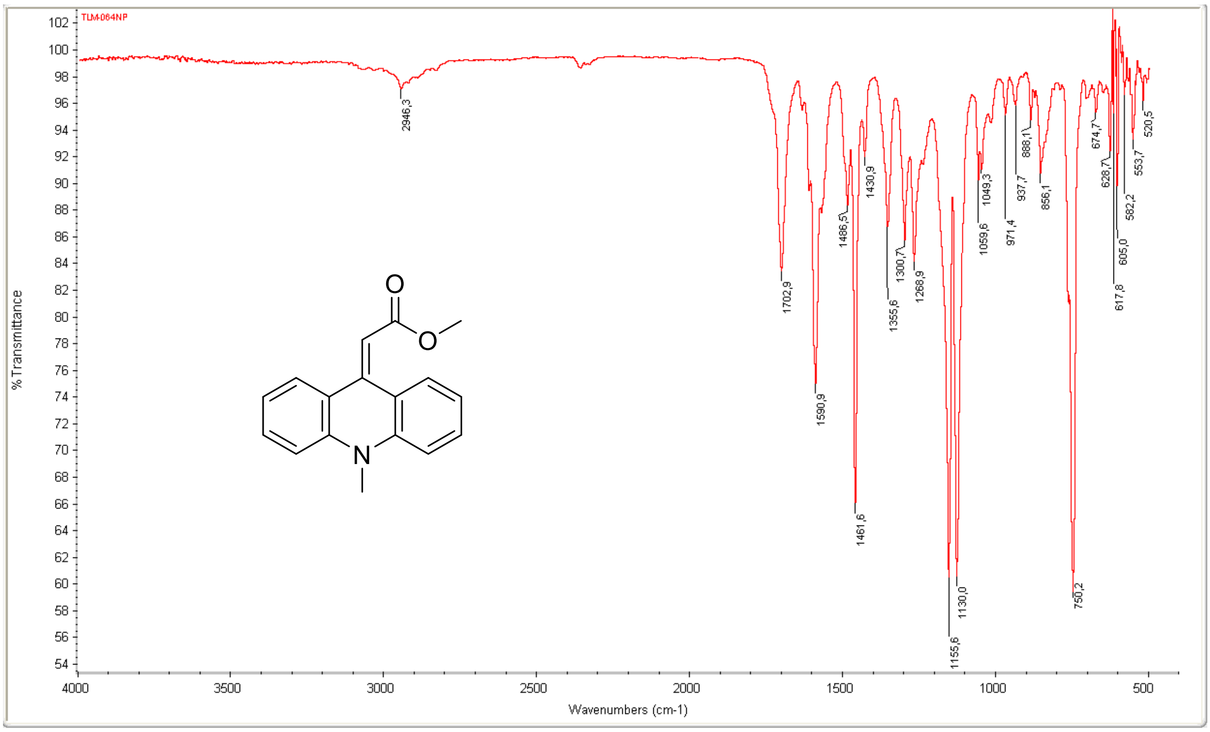

Figure S2: NMR- and IR-spectra of **8**.

1.3 NMR and IR spectra of compound 10

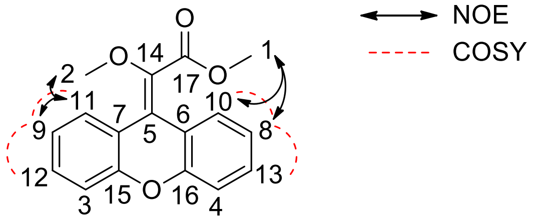

Table S3: 1D and 2D-NMR data of Methyl 2-methoxy-2-(9H-xanthen-9-ylidene)acetate (10) in $CDCl_3$, at 298 K and 500 MHz for ^{1}H and 125 MHz for ^{13}C.

No.	δ_H (*J* in Hz)	δ_C, mult.	HMBC (^xJ)
1	3.74 (3H, s)	51.9, CH_3	-
2	3.59 (3H, s)	57.6, CH_3	-
3	7.19 (1H, m)	116.2, CH_{arom}	C3→H9(^3J)
4	7.19 (1H, m)	116.5, CH_{arom}	C4→H8(^3J)
5		117.1, C_q	C5→H10(^3J), H11(^3J)
6		121.0, C_q	C6→H4(^3J), H8(^3J)
7		121.4, C_q	C7→H3(^3J), H9(^3J)
8	7.05 (1H, ddd, 7.9/7.2/1.3 Hz)	122.8, CH_{arom}	C8→H4(^3J), H13(^2J)
9	7.15 (1H, m)	123.1, CH_{arom}	C9→H3(^3J), H11(^2J)
10	7.21 (1H, m)	126.7, CH_{arom}	C10→H8(^2J), H13(^3J)
11	8.15 (1H, dd, 8.0/1.6 Hz)	128.7, CH_{arom}	C11→H12(^3J)
12	7.30 (1H, m)	128.9, CH_{arom}	C12→H11(^3J)
13	7.30 (1H, m)	129.1, CH_{arom}	C13→H10(^3J)
14		140.9, C_q	C14→H12(^3J)
15		152.3, C_q	C15→H11(^3J), H12(^3J)
16		152.6, C_q	C16→H8(^4J), H10(^3J), H13(^3J)
17		165.9, C_q	C17→H1(^3J)

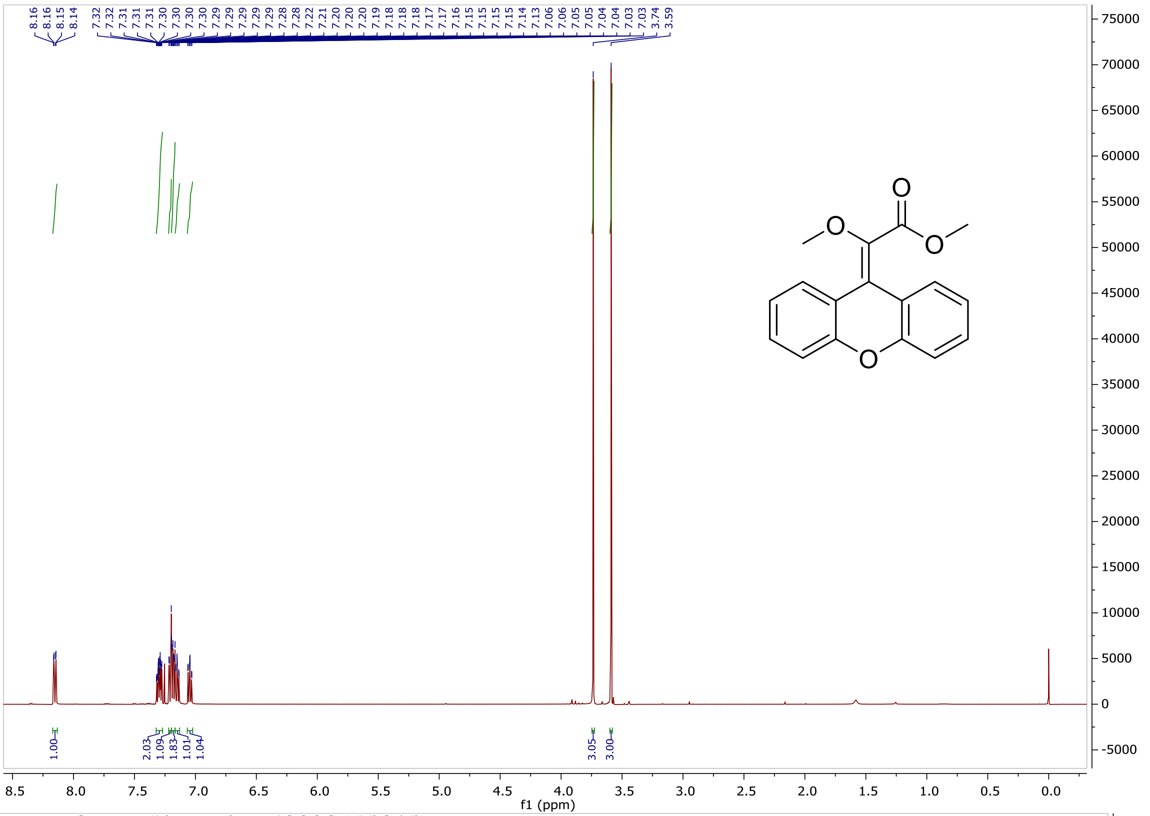
8.16
8.16
8.15
8.14
7.32
7.32
7.31
7.31
7.31
7.30
7.30
7.30
7.30
7.30
7.29
7.29
7.29
7.29
7.29
7.28
7.28
7.22
7.21
7.20
7.20
7.20
7.19
7.18
7.18
7.18
7.17
7.17
7.16
7.15
7.15
7.15
7.15
7.14
7.13
7.06
7.06
7.05
7.05
7.04
7.04
7.03
7.03
3.74
3.59
1.00
2.03
1.09
1.83
1.01
1.04
3.05
3.00
f1 (ppm)

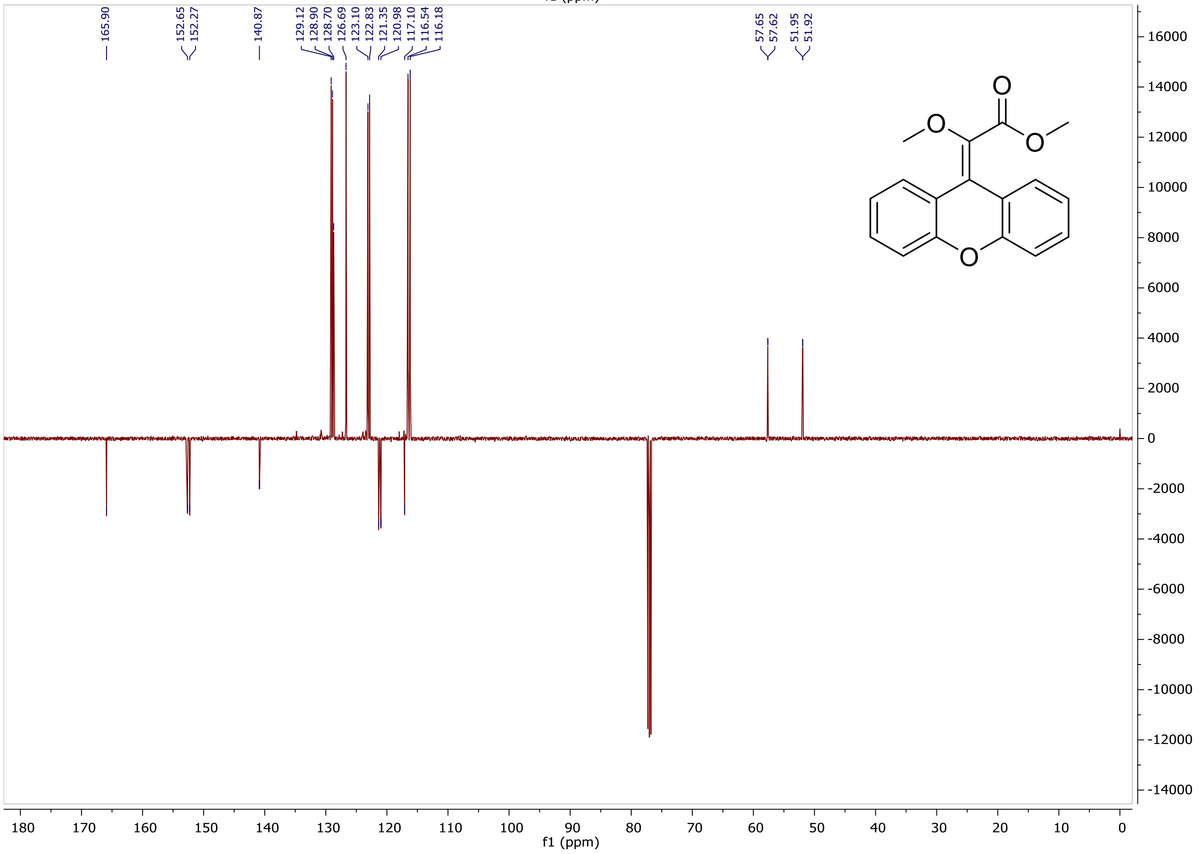
165.90
152.65
152.27
140.87
129.12
128.90
128.70
126.69
123.10
122.83
121.35
120.98
117.10
116.54
116.18
57.65
57.62
51.95
51.92
f1 (ppm)

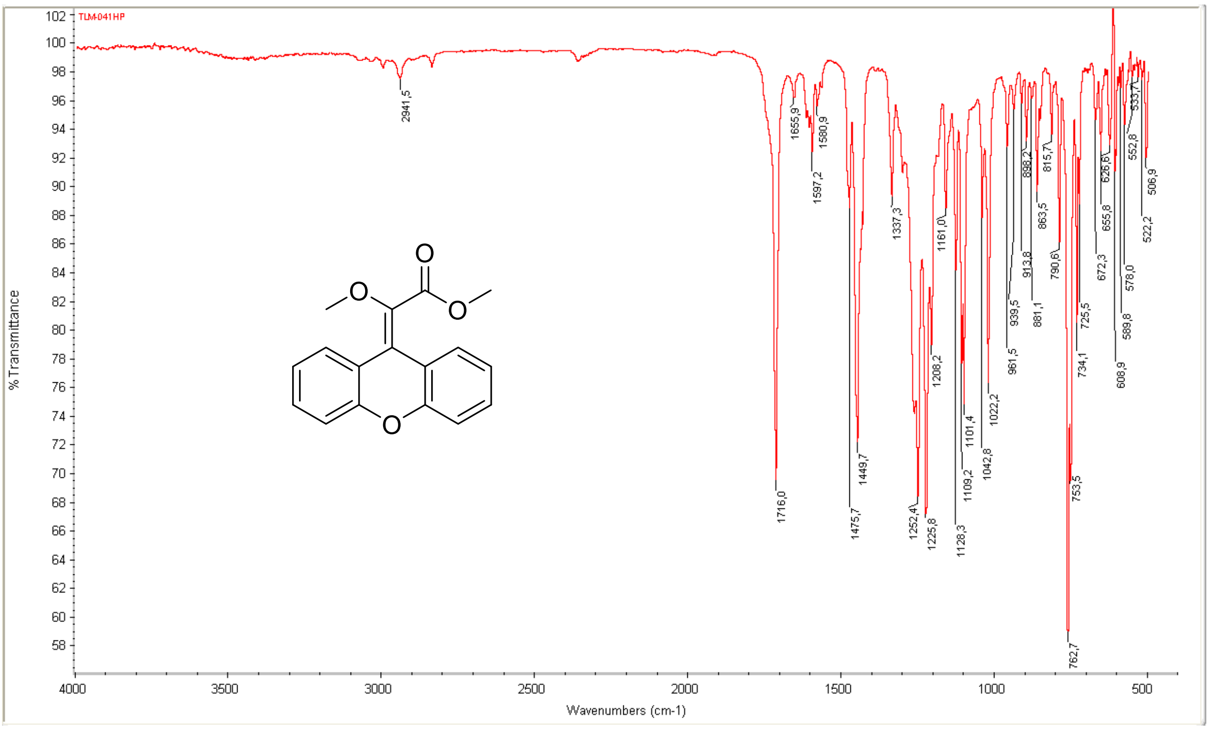

<u>Figure S3:</u> NMR- and IR-spectra of **10**.

1.4 NMR and IR spectra of compound 11

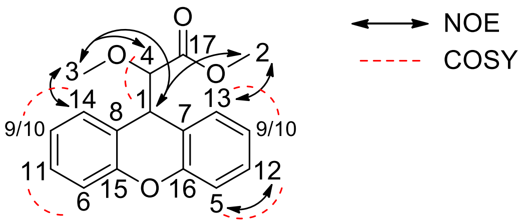

Table S4: 1D and 2D-NMR data of Methyl 2-methoxy-2-(9H-xanthen-9-yl)acetate (11) in $CDCl_3$, at 298 K and 500 MHz for ^1H and 125 MHz for ^{13}C.

No.	δ_H (*J* in Hz)	δ_C, mult.	HMBC (^xJ)
1	4.40 (1H, d, 5.6 Hz)	43.1, CH	C1→H4(^2J), H5(^4J), H6(^4J), H13(^3J), H14(^3J)
2	3.60 (3H, s)	51.8, CH_3	-
3	3.21 (3H, s)	59.0, CH_3	C3→H4(^3J)
4	3.83 (1H, d, 5.6 Hz)	86.5, CH	C4→H1(^2J), H3(^3J)
5	7.12 (1H, d, 7.9 Hz)	116.4, CH_{arom}	C5→H9/H10(^3J)
6	7.11 (1H, d, 7.9 Hz)	116.5, CH_{arom}	C6→H9/H10(^3J)
7		120.8, C_q	C7→H1(^2J), H4(^3J), H5(^3J), H9/H10(^3J)
8		121.0, C_q	C8→H1(^2J), H4(^3J). H6(^3J), H9/H10(^3J)
9	7.07 (1H, tt, 7.5/1.7 Hz)	123.0, CH_{arom}	C9→H5/H6(^3J)
10	7.07 (1H, tt, 7.5/1.7 Hz)	123.2, CH_{arom}	C10→H5/H6(^3J)
11	7.28 (1H, m)	128.4, CH_{arom}	C11→H14(^3J)
12	7.26 (1H, m)	128.5, CH_{arom}	C12→H13(^3J)
13	7.24 (1H, m)	128.8, CH_{arom}	C13→H1(^3J), H9/H10(^2J)
14	7.22 (1H, dd, 7.6/1.4 Hz)	129.4, CH_{arom}	C14→H1, H9/H10(^2J)
15		152.8, C_q	C15→H1(^3J), H6(^2J), , H11(^3J), H14(^3J)
16		153.0, C_q	C16→H1(^3J), H5(^2J), H11(^3J), H14(^3J)
17		171.2, C_q	C17→H1(^3J), H2(^3J), H4(^2J)

Note: Carbon 9/10 could not be assigned completely as they are very similar in the 2D-NMR-spectra.

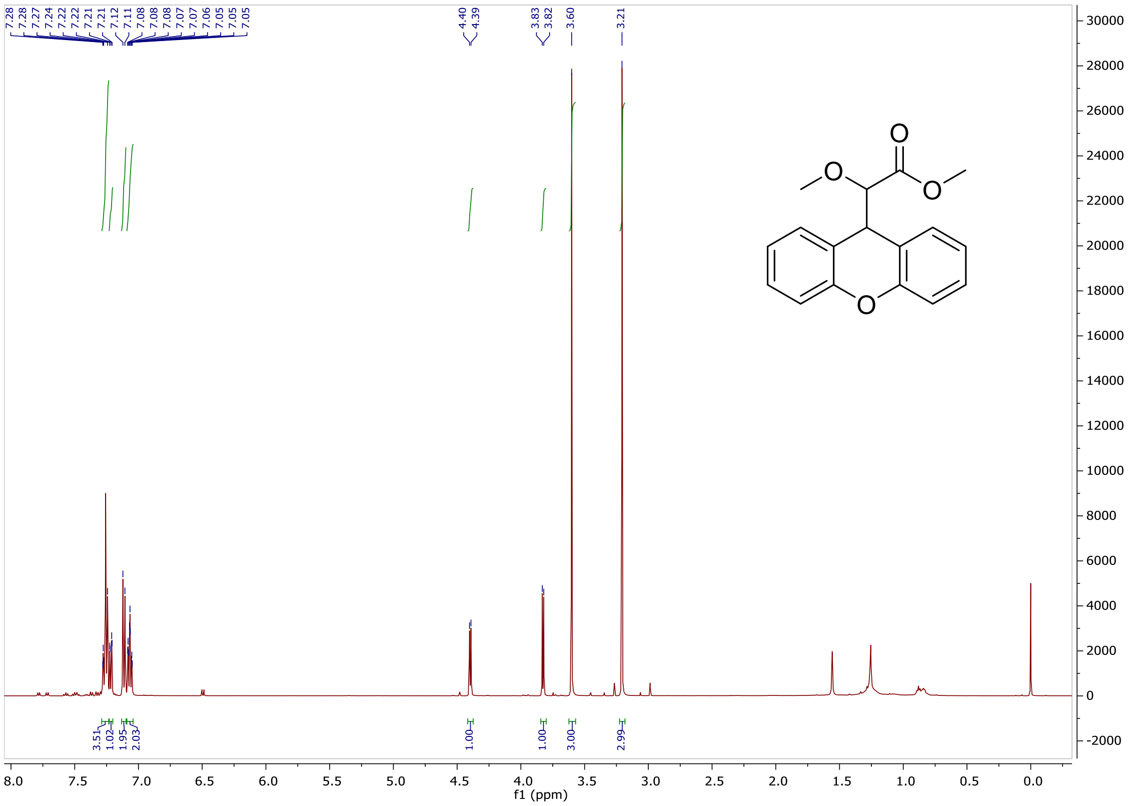
7.28
7.28
7.27
7.24
7.22
7.22
7.21
7.21
7.12
7.11
7.08
7.08
7.08
7.07
7.07
7.06
7.05
7.05
7.05
4.40
4.39
3.83
3.82
3.60
3.21
3.51
1.02
1.95
2.03
1.00
1.00
3.00
2.99
f1 (ppm)

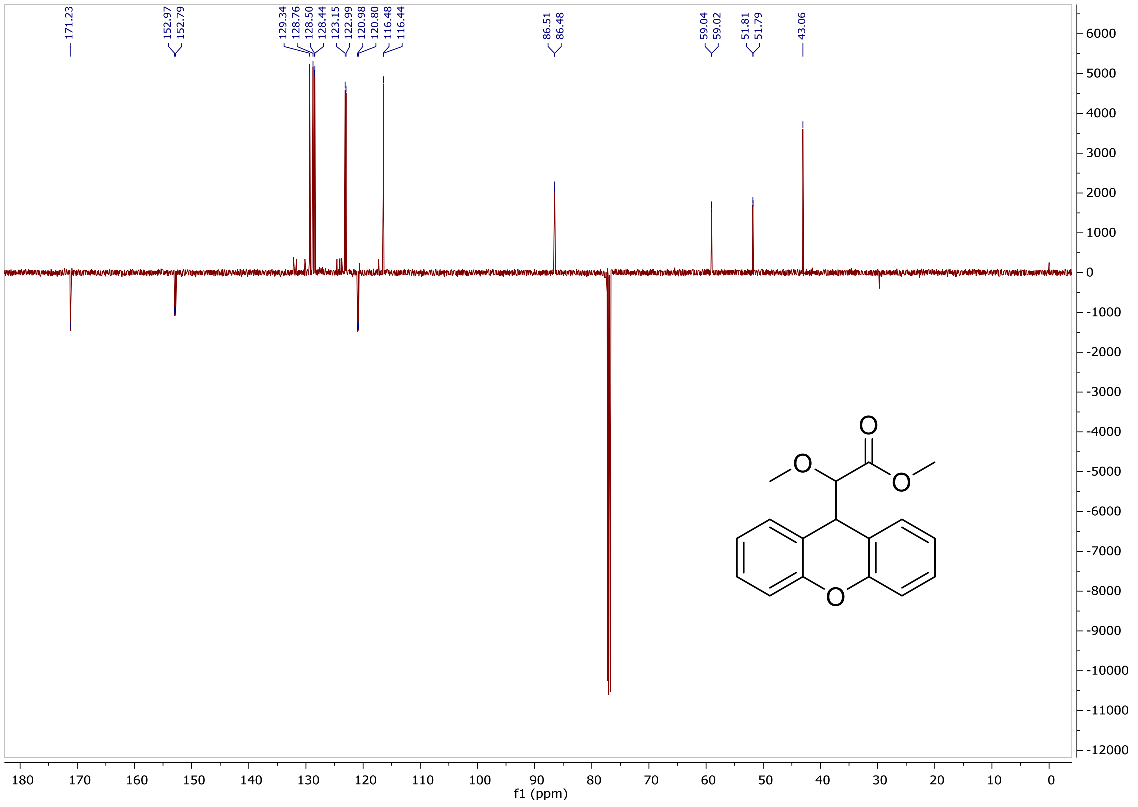
171.23
152.97
152.79
129.34
128.76
128.50
128.44
123.15
122.99
120.98
120.80
116.48
116.44
86.51
86.48
59.04
59.02
51.81
51.79
43.06
f1 (ppm)

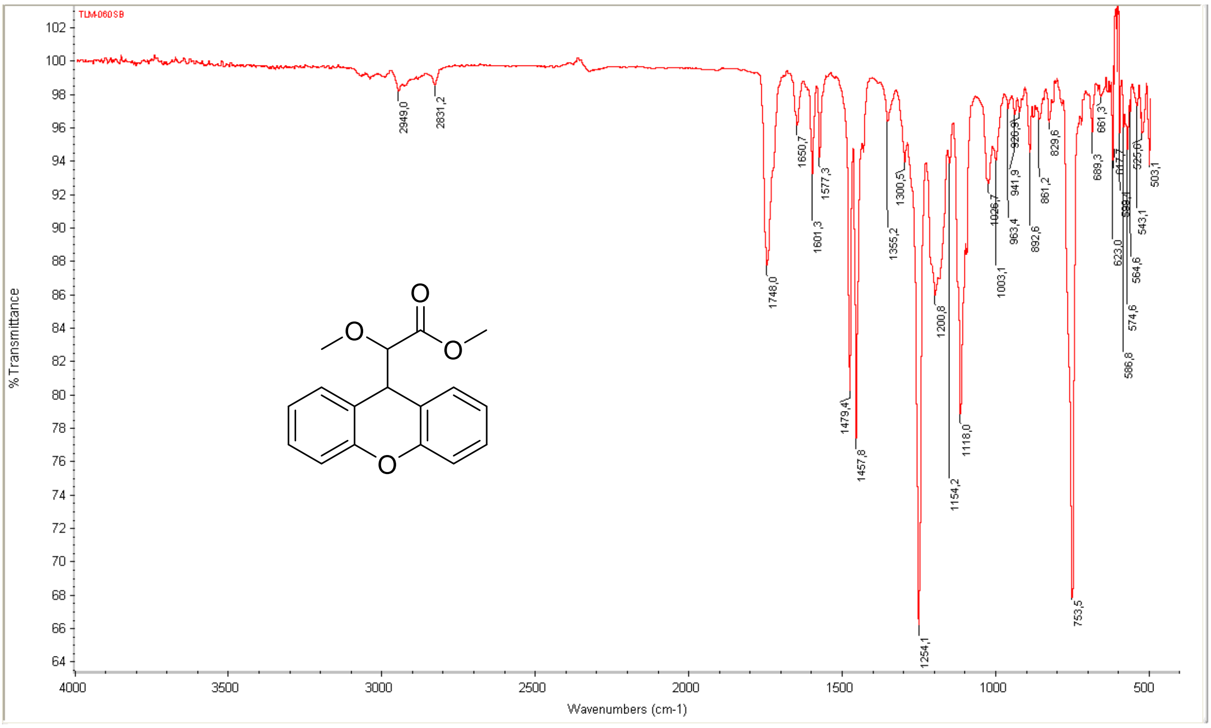

Figure S4: NMR- and IR-spectra of **10**.

1.5 NMR and IR spectra of compound 14

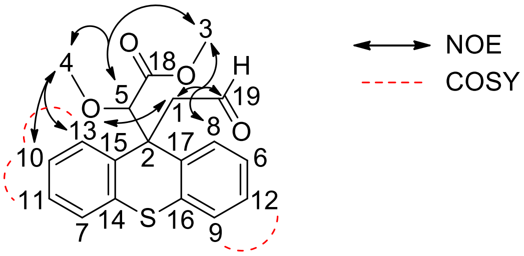

Table S5: 1D and 2D-NMR data of Methyl 2-methoxy-2-(9-(2-oxoethyl)-9H-thioxanthen-9-yl)acetate (14) in $CDCl_3$, at 298 K and 500 MHz for ^1H and 125 MHz for ^{13}C.

No.	δ_H (*J* in Hz)	δ_C, mult.	HMBC (^xJ)
1_a	3.71 (1H, dd, 19.7/2.3 Hz)	40.5, CH_2	C1→H5(^3J), H19(^2J)
1_b	3.97 (1H, dd, 19.7/1.9 Hz)	40.5, CH_2	C1→H5(^3J), H19(^2J)
2		48.9, C_q	C2→$H1_{a,b}$(^2J), H5(^2J), H8(^3J), H13(^3J), H19(^3J)
3	3.27 (3H, s)	51.6, CH_3	-
4	2.99 (3H, s)	58.9, CH_3	C4→H5(^3J)
5	4.48 (1H, s)	78.5, CH	C5→$H1_{a,b}$(^3J), H4(^3J)
6	7.30 (1H, m)	126.5, CH_{arom}	C6→H9(^3J)
7	7.22 (1H, m)	126.6, CH_{arom}	C7→H11(^2J)
8	7.19 (1H, m)	127.1, CH_{arom}	C8→H6(^2J), H12(^3J)
9	7.48 (1H, m)	127.2, CH_{arom}	C9→H12(^2J)
10	7.29 (1H, m)	127.3, CH_{arom}	C10→H7(^3J)
11	7.48 (1H, m)	127.4, CH_{arom}	C11→H13(^3J)
12	7.26 (1H, m)	127.7, CH_{arom}	C12→H8(^3J)
13	7.41 (1H, m)	128.9, CH_{arom}	C13→H10(^3J)
14		131.9, C_q	C14→H10(^4J), H13(^3J)
15		133.1, C_q	C15→$H1_{a,b}$(^3J), H5(^3J), H7(^3J), H11(^4J)
16		133.3, C_q	C16→H8(^3J), H12(^3J)
17		134.4, C_q	C17→$H1_{a,b}$(^3J), H5(^3J), H6(^3J), H9(^4J), H13(^4J)
18		170.6, C_q	C18→H3(^2J), H5(^2J)
19	9.61 (1H, t, 2.0 Hz)	202.1, $C_{aldheyde}$	C19→ $H1_{a,b}$(^3J)

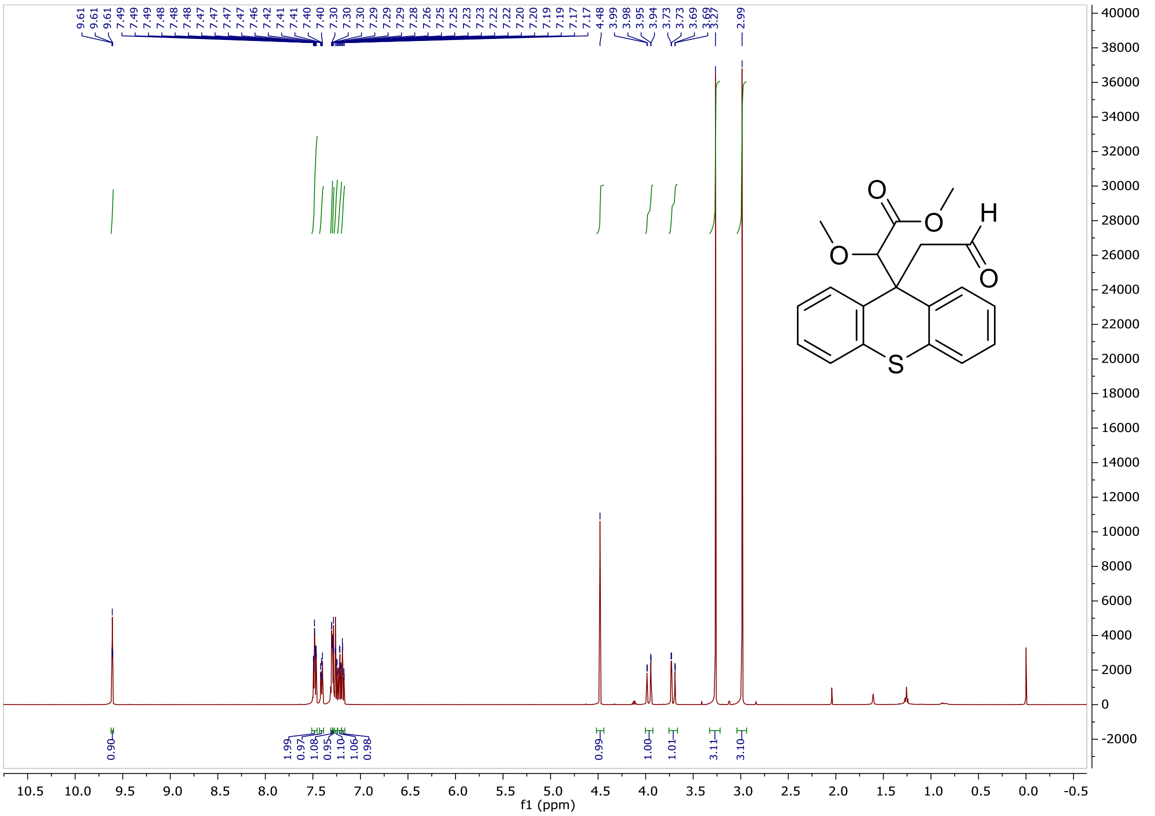
9.61
9.61
9.61
7.49
7.49
7.48
7.48
7.48
7.47
7.47
7.47
7.46
7.42
7.41
7.41
7.40
7.40
7.30
7.30
7.30
7.29
7.29
7.29
7.28
7.26
7.25
7.25
7.23
7.23
7.22
7.22
7.20
7.20
7.19
7.19
7.17
7.17
4.48
3.99
3.98
3.95
3.94
3.73
3.73
3.69
3.69
3.27
2.99
0.90
1.99
0.97
1.08
0.95
1.10
1.06
0.98
0.99
1.00
1.01
3.11
3.10
f1 (ppm)
O
O
H
O
O
S

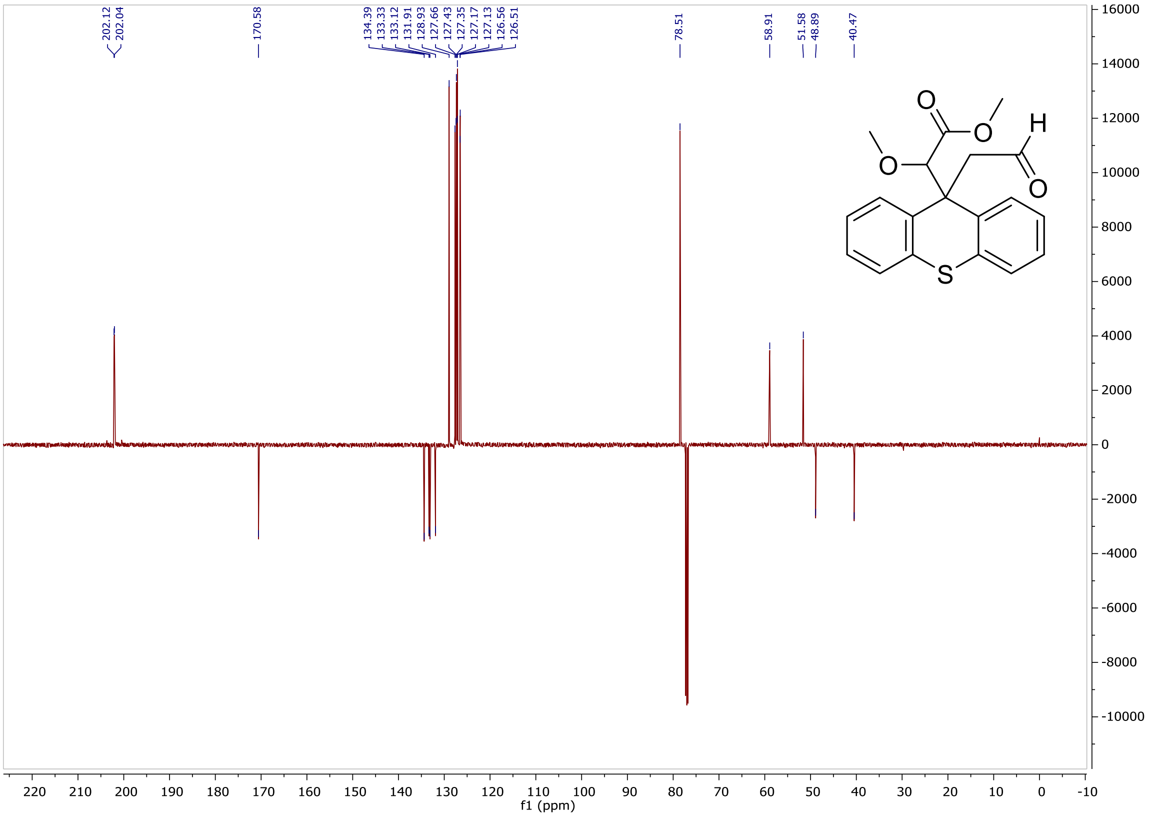
202.12
202.04
170.58
134.39
133.33
133.12
131.91
128.93
127.66
127.43
127.35
127.17
127.13
126.56
126.51
78.51
58.91
51.58
48.89
40.47
f1 (ppm)
O
O
H
O
O
S

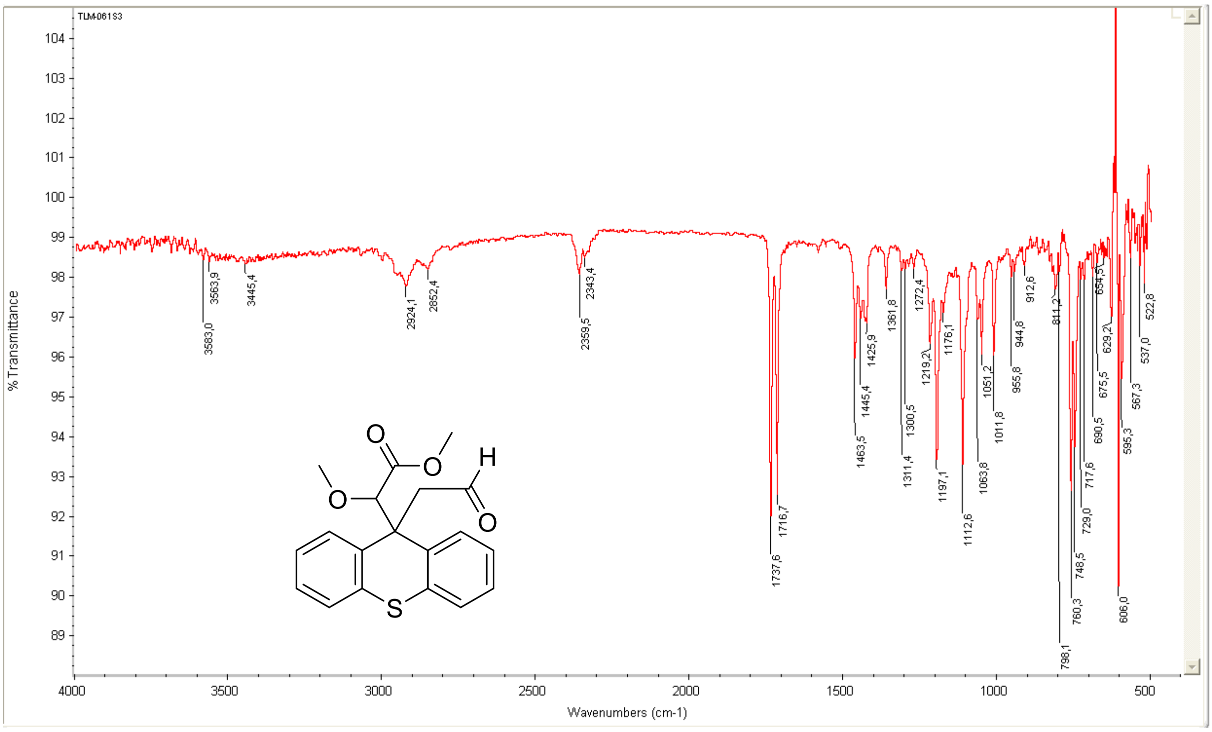

Figure S5: NMR- and IR-spectra of **14**.

1.6 NMR and IR spectra of compound 15

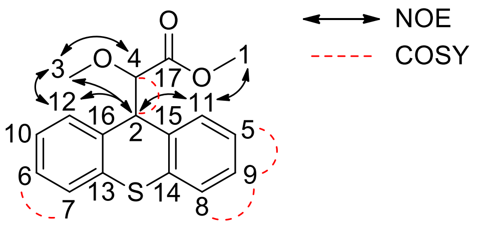

<u>Table S6:</u> 1D and 2D-NMR data of Methyl 2-methoxy-2-(9H-thioxanthen-9-ylidene)acetate (15) in $CDCl_3$, at 298 K and 600 MHz for ^{1}H and 150 MHz for ^{13}C.

No.	δ_H (J in Hz)	δ_C, mult.	HMBC (^xJ)
1	3.42 (3H, s)	51.6, CH_3	-
2	4.36 (1H, d, 9.6 Hz)	52.6, CH	C2→H4(^2J), H7(^4J), H8(^4J), H11(^3J), H12(^3J)
3	3.10 (3H, s)	58.6, CH_3	C3→H4(^3J)
4	4.13 (1H, d, 9.7 Hz)	79.0, CH	C4→H2(^2J), H3(^3J)
5	7.17 (1H, m)	126.3, CH_{arom}	C5→H8(^3J)
6	7.25 (1H, m)	126.4, CH_{arom}	C6→H7(^2J), H12(^3J)
7	7.42 (1H, m)	126.6, CH_{arom}	C7→H6(^2J), H10(^3J)
8	7.42 (1H, m)	126.9, CH_{arom}	C8→H5(^3J)
9	7.23 (1H, m)	127.2, CH_{arom}	C9→H11(^3J)
10	7.21 (1H,m)	127.4, CH_{arom}	C10→ H7(^3J), H12(^2J)
11	7.19 (1H, m)	129.4, CH_{arom}	C11→H2(^3J), H9(^3J)
12	7.40 (1H, m)	131.3, CH_{arom}	C12→H2(^3J), H6(^3J)
13		132.2, C_q	C13→H2(^3J), H7(^2J), H12(^3J)
14		132.8, C_q	C14→H2(^3J), H8(^2J), H9(^3J)
15		133.4, C_q	C15→H2(^2J), H4(^3J), H9(^4J), H11(^2J)
16		134.1, C_q	C16→H2(^2J), H4(^3J), H12(^2J)
17		171.8, C_q	C17→H1(^3J), H2(^3J), H4(^2J)

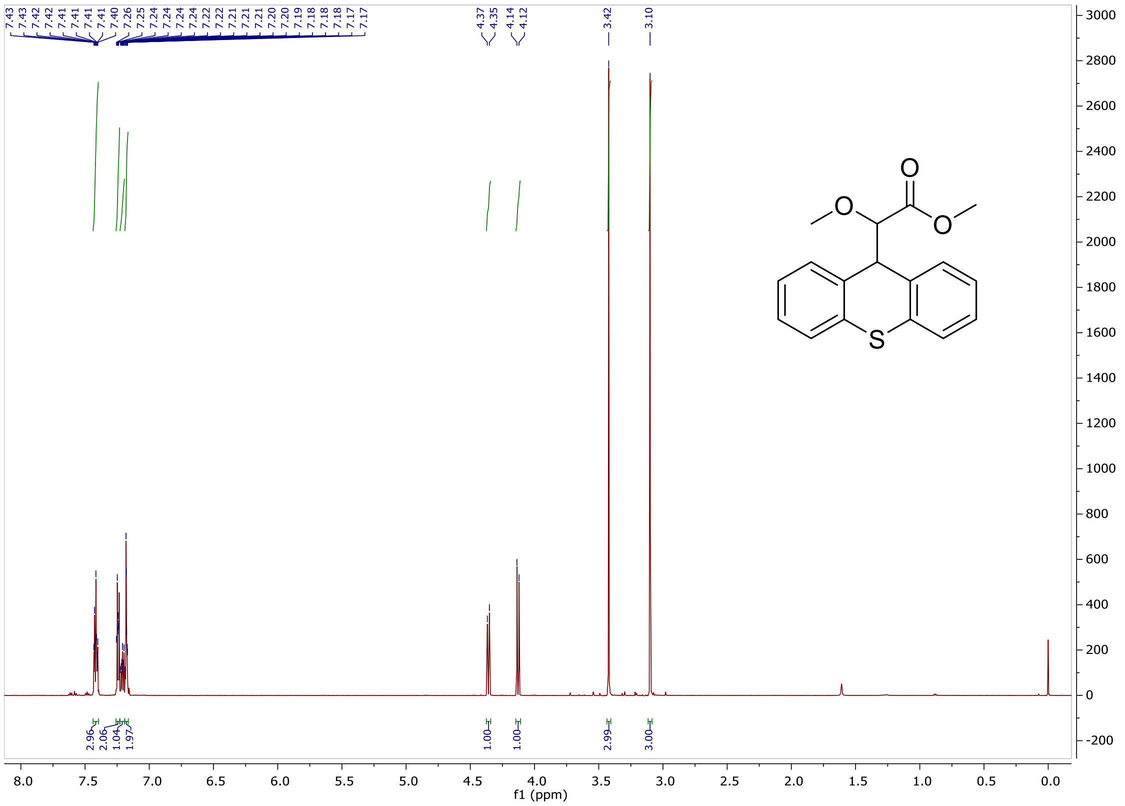
7.43
7.43
7.42
7.42
7.41
7.41
7.41
7.41
7.40
7.26
7.25
7.24
7.24
7.24
7.24
7.22
7.22
7.21
7.21
7.21
7.20
7.20
7.19
7.18
7.18
7.18
7.17
7.17
4.37
4.35
4.14
4.12
3.42
3.10
2.96
2.06
1.04
1.97
1.00
1.00
2.99
3.00
O
O
O
S
f1 (ppm)

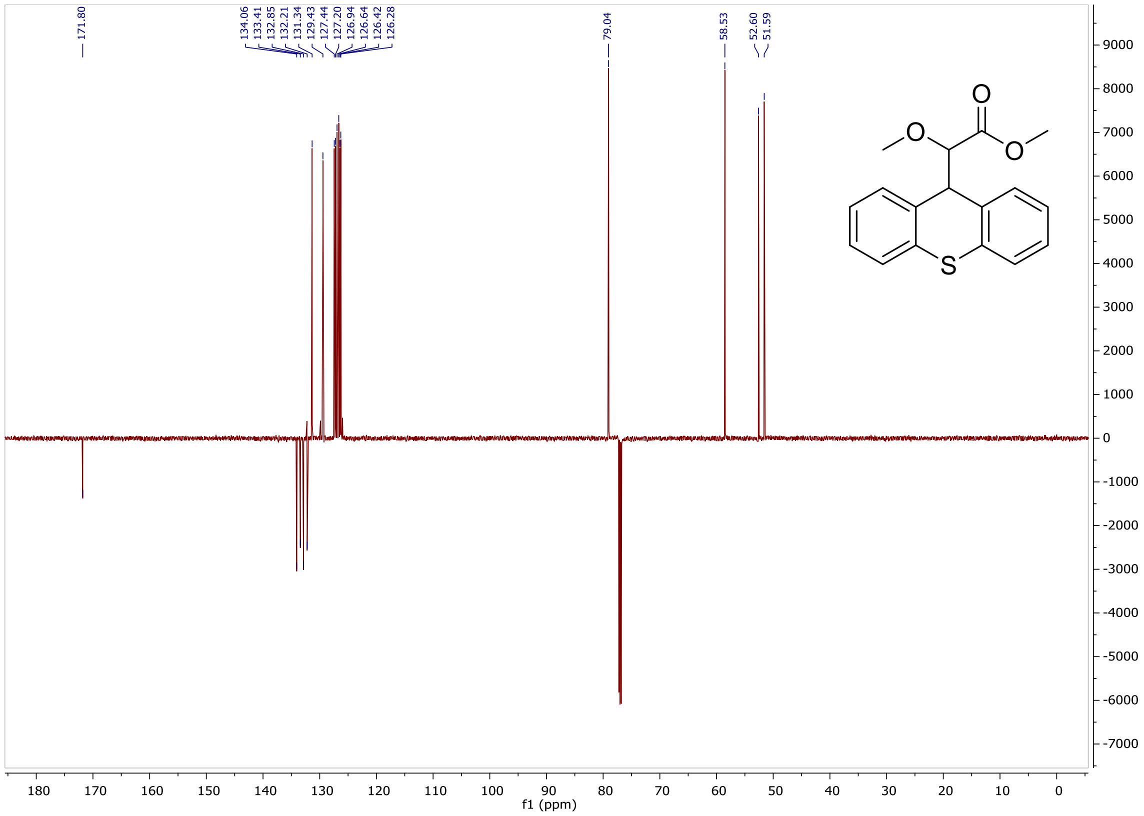
171.80
134.06
133.41
132.85
132.21
131.34
129.43
127.44
127.20
126.94
126.64
126.42
126.28
79.04
58.53
52.60
51.59
O
O
O
S
f1 (ppm)

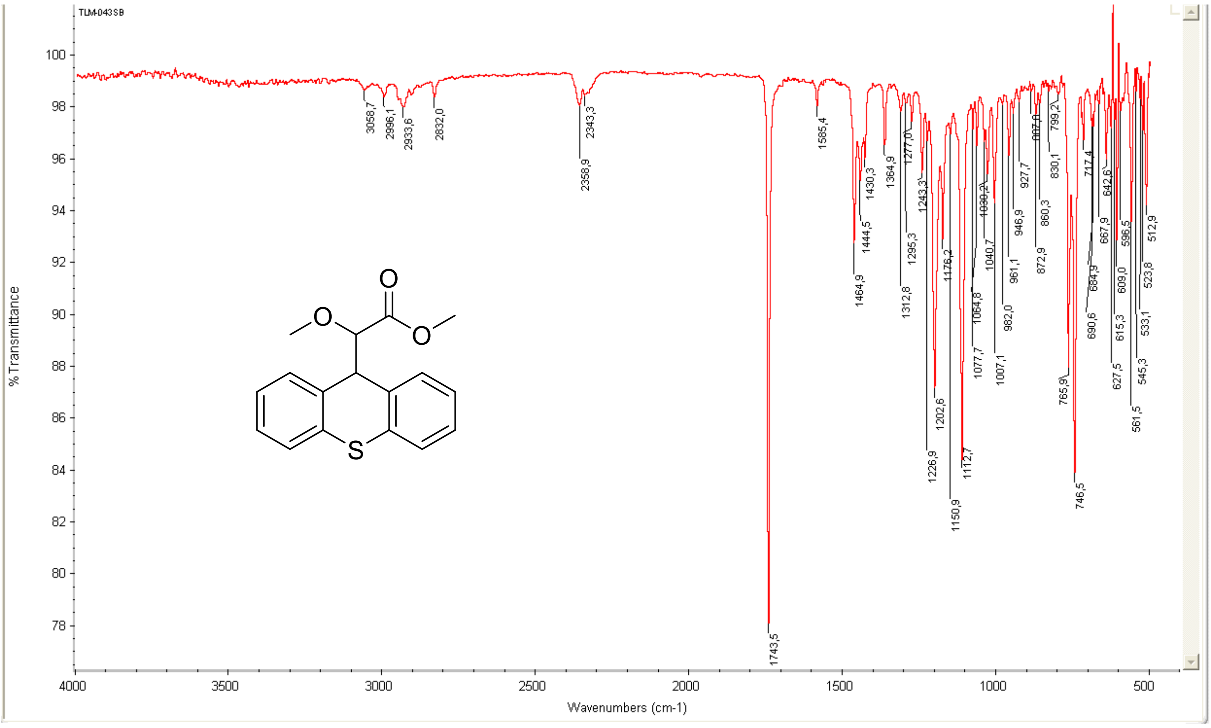

Figure S6: NMR- and IR-spectra of **15**.

2. X-ray data

2.1 Data of X-ray crystal measurement of 14

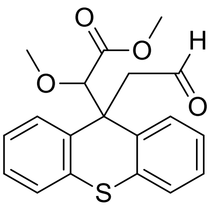

14

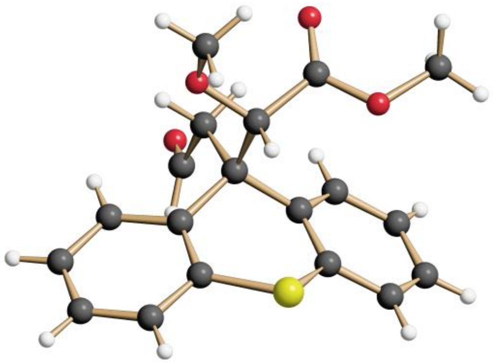

Table S7: Crystal data and structure refinement of **14**.

Moiety formula	$C_{19}H_{18}O_4S$
Formula weight	342.39
Temperature	100(2) K
Wavelength	1.54178 Å
Crystal system	Monoclinic
Space group	$P2_1/c$
Unit cell dimensions	a = 12.4141(4) Å
	b = 8.8021(3) Å
	c = 15.1236(4) Å
	ß = 98.8078(14)°
Volume	1633.07(9) $Å^3$
Z	4
Density (calculated)	1.393 Mg/m^3
Absorption coefficient	1.936 mm^{-1}
F(000)	720
Crystal size	0.300 x 0.150 x 0.030 mm^3
Index ranges	-15<=h<=13, -10<=k<=10, -18<=l<=18
Collected / Independent reflections	24579 / 3218 [R(int) = 0.0670]
Data / restraints / parameters	3218 / 0 / 219
Goodness-of-fit on F^2	1.029
Final R indices [I>2sigma(I)]	R1 = 0.0336, wR2 = 0.0724
R indices (all data)	R1 = 0.0436, wR2 = 0.0763
Largest diff. peak and hole	0.298 and -0.286 e.$Å^{-3}$

2.2 Data of X-ray crystal measurement of 15

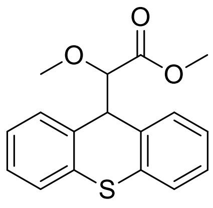

15

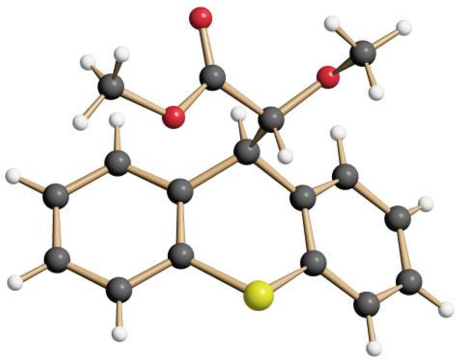

<u>Table S8:</u> Crystal data and structure refinement of **15**.

Moiety formula	$C_{17}H_{16}O_3S$
Formula weight	300.36
Temperature	100(2) K
Wavelength	1.54178 Å
Crystal system	Monoclinic
Space group	C2/c
Unit cell dimensions	a = 15.4139(9) Å
	b = 8.7429(5) Å
	c = 21.8769(13) Å
	ß = 102.736(2)°
Volume	2875.6(3) $Å^3$
Z	8
Density (calculated)	1.388 Mg/m^3
Absorption coefficient	2.064 mm^{-1}
F(000)	1264
Crystal size	0.200 x 0.200 x 0.150 mm^3
Index ranges	-18<=h<=18, -10<=k<=10, -22<=l<=27
Collected / Independent reflections	13063 / 2798 [R(int) = 0.0442]
Data / restraints / parameters	2798 / 0 / 192
Goodness-of-fit on F^2	1.086
Final R indices [I>2sigma(I)]	R1 = 0.0458, wR2 = 0.1227
R indices (all data)	R1 = 0.0475, wR2 = 0.1240
Largest diff. peak and hole	0.577 and -0.489 $e.Å^{-3}$
